# Combined Targeting of AKT and mTOR Inhibits Tumor Formation of EpCAM^+^ and CD90^+^ Human Hepatocellular Carcinoma Cells in an Orthotopic Mouse Model

**DOI:** 10.3390/cancers14081882

**Published:** 2022-04-08

**Authors:** Mohamed Moustafa, Katarzyna-Krystyna Dähling, Armin Günther, Leonie Riebandt, Daniel J. Smit, Kristoffer Riecken, Carina Schröder, Ruimeng Zhuang, Till Krech, Malte Kriegs, Boris Fehse, Jakob R. Izbicki, Lutz Fischer, Björn Nashan, Jun Li, Manfred Jücker

**Affiliations:** 1 Institute of Biochemistry and Signal Transduction, Center for Experimental Medicine, University Medical Center Hamburg-Eppendorf, 20246 Hamburg, Germany; dr_ezzo81@hotmail.com (M.M.); katarzyna-k.daehling@stud.uke.uni-hamburg.de (K.-K.D.); armin.guenther@uni-hamburg.de (A.G.); leonieriebandt@yahoo.de (L.R.); d.smit@uke.de (D.J.S.); carina.schroeder@tum.de (C.S.); zhuang.ruimeng@stud.uke.uni-hamburg.de (R.Z.); 2 Research Department Cell and Gene Therapy, Department of Stem Cell Transplantation, University Medical Center Hamburg-Eppendorf, 20246 Hamburg, Germany; k.riecken@uke.uni-hamburg.de (K.R.); fehse@uke.de (B.F.); 3 Department of Pathology, University Medical Center Hamburg-Eppendorf, 20246 Hamburg, Germany; t.krech@uke.de; 4 Department of Radiotherapy & Radiation Oncology, University Medical Center Hamburg-Eppendorf, 20246 Hamburg, Germany; m.kriegs@uke.de; 5 Department of General, Visceral and Thoracic Surgery, University Medical Center Hamburg-Eppendorf, 20246 Hamburg, Germany; izbicki@uke.de (J.R.I.); j.li@uke.de (J.L.); 6 Department of Visceral Transplant Surgery, University Medical Center Hamburg-Eppendorf, 20246 Hamburg, Germany; lutz.fischer@uke.de; 7 Clinic for Hepatopancreaticobiliary Surgery and Transplantation, First Affiliated Hospital, University of Science and Technology of China, Hefei 230001, China; nashan@ustc.edu.cn

**Keywords:** hepatocellular carcinoma, AKT, mTOR, combined treatment, tumor spheroids, orthotopic mouse model, treatment resistance, EpCAM, CD90, CSC

## Abstract

**Simple Summary:**

Cancer stem cells are a distinct tumor subpopulation associated with poor outcome. The epithelial cell adhesion molecule (EpCAM) and Thy-1 cell surface antigen (CD90) have been implicated as cancer stem cell (CSC) markers in hepatocellular carcinoma (HCC). Eradication of those CSCs is of high importance with respect to the outcome of treatment. We aimed to verify the impact of targeting HCC cells expressing the CSC marker CD90 and EpCAM with combined therapy of AKT and mTOR inhibitors. Our data demonstrated that combined targeting of AKT and mTOR is highly synergistic in vitro but leads to treatment resistance of xenograft tumors in an orthotopic mouse model in vivo. The development of resistance was observed in large numbers of MK2206/RAD001 treated cells. Restoration of phosphorylated AKT was observed in most tumors during AKT/mTOR therapy underlining the importance of restored AKT signaling as a resistance mechanism. Further work is required to verify the molecular mechanisms of HCC treatment resistance.

**Abstract:**

The epithelial cell adhesion molecule (EpCAM) and Thy-1 cell surface antigen (CD90) have been implicated as cancer stem cell (CSC) markers in hepatocellular carcinoma (HCC). Expression of EpCAM and CD90 on HCC cells is associated with increased tumorigenicity, metastasis and poor prognosis. In this study, we demonstrate that combined treatment with AKT and mTOR inhibitors—i.e., MK2206 and RAD001—results in a synergistic reduction in proliferation of EpCAM^+^ and CD90^+^ HCC cells cultured either as adherent cells or as tumoroids in vitro. In addition, tumor growth was reduced by combined treatment with AKT and mTOR inhibitors in an orthotopic xenograft mouse model of an EpCAM^+^ HCC cell line (Huh7) and primary patient-derived EpCAM^+^ HCC cells (HCC1) as well as a CD90^+^ HCC-related cell line (SK-HEP1) in vivo. However, during AKT/mTOR treatment, outgrowth of therapy-resistant tumors was observed in all mice analyzed within a few weeks. Resistance was associated in most cases with restoration of AKT signaling in the tumors, intrahepatic metastases and distant metastases. In addition, an upregulation of the p38 MAPK pathway was identified in the AKT/mTOR inhibitor-resistant tumor cells by kinome profiling. The development of resistant cells during AKT/mTOR therapy was further analyzed by red-green-blue (RGB) marking of HCC cells, which revealed an outgrowth of a large number of Huh7 cells over a period of 6 months. In summary, our data demonstrate that combined treatment with AKT and mTOR inhibitors exhibits synergistic effects on proliferation of EpCAM^+^ as well as CD90^+^ HCC cells in vitro. However, the fast development of large numbers of resistant clones under AKT/mTOR therapy observed in vitro and in the orthotopic xenotransplantation mouse model in vivo strongly suggests that this therapy alone will not be sufficient to eliminate EpCAM^+^ or CD90^+^ cancer stem cells from HCC patients.

## 1. Introduction

Hepatocellular carcinoma (HCC) constitutes more than 90% of primary liver tumors. It is considered a major global health problem, as it is the 6th most prevalent cancer and the second leading cause of cancer-related mortality worldwide [1,2,3]. Liver transplantation and surgical resection remain the gold standard for curative treatment of HCC, with an average median overall survival (OS) of more than 5 years. Patients with advanced stages, due to extensive tumor burden, or inoperable, due to performance status, are offered other treatment modalities, including systemic therapy [4,5,6]. Targeted therapy strategies in HCC were extensively examined in the past. Especially, the group of (receptor) tyrosine kinase inhibitors including sorafinib, sunitinib or cetuximab for HCC patients was extensively studied in the past [7]. In 2007, sorafenib, a multikinase inhibitor, was approved by the FDA as a unique targeted drug for advanced HCC. With a modest efficacy in increasing survival (OS of 10 months), sorafenib remains the first line of systemic treatment in the current guidelines [8,9]. In addition to targeted treatment with tyrosine kinase inhibitors, immune checkpoint therapies were also approved by the FDA (e.g., nivolumab, pembrolizumab) [10]. However, to date, no other investigated potential drugs (e.g., sunitinib, erlotinib) including systemic chemotherapeutic agents (e.g., doxorubicin or the FOLFOX regimen) demonstrated a superior survival benefit over sorafenib in advanced HCC patients [11,12,13,14]. However, sorafenib has been associated with some adverse side effects and drug resistance [15,16]. Hence comes the need for new effective treatment strategies, which may increase the quality-adjusted-years of life.

A major problem for a successful treatment of cancer is the heterogeneity of the tumor cells [17,18]. The concept of tumor progression in a hierarchical model, generated by a minority of tumor cells known as cancer stem cells (CSCs), has been widely accepted and helped to reveal the initiation and diversity of tumor cells within cancer tissue. CSCs harbor the capacity of self-renewal and pluripotency, account for the tumor progression, metastasis and recurrence of cancers and are more resistant against chemo- and radiotherapy. Therefore, CSCs are considered as a pivotal target for eradicating cancer [19,20]. Many studies have supported the evidence regarding the origin of CSCs in human HCC. CSCs have been isolated from primary HCC and from HCC cell lines [21,22,23,24]. Recent studies reported that CD90^+^ CSCs are highly metastatic and are associated with a poor prognosis [25,26,27].

The PI3K/AKT/mTOR signaling pathway has been under the spotlight of oncology researchers for a long time. It has been well documented that activation of this signaling pathway was frequently detected in an immunohistochemical analysis of HCC tissue samples [28]. In addition, activation of mammalian target of rapamycin (mTOR) appears to be associated with less differentiated tumors, poor survival and early recurrence after surgical resection and an impaired overall prognosis in advanced HCC [29,30,31,32]. Several inhibitors targeting mTOR were tested in clinical trials. A preliminary antitumor activity was observed in a phase I/II study of mTOR inhibition using the rapamycin derivate RAD001 (everolimus) in advanced HCC. However, in a subsequent phase III trial (EVOLVE-1), which included 546 patients with advanced HCC patients, whose disease progressed during or after receiving sorafenib or who were intolerant to sorafenib, single-agent therapy with everolimus did not improve the overall survival, and the trial did not meet its primary endpoint [33,34,35]. Several cellular mechanisms have been described, which might limit the therapeutic effects of RAD001, including a negative feedback loop resulting in the activation of AKT following mTOR inhibition [36,37]. 

In order to counteract the negative feedback activation of AKT after mTOR inhibition, we analyzed the combined effects of AKT and mTOR inhibitors on HCC cell lines in vitro and in vivo. We demonstrated that dual targeting of AKT and mTOR has a synergistic effect on the inhibition of HCC cell proliferation in vitro as well as on tumor growth and survival in a subcutaneous xenotransplantation mouse model in vivo [38]. Similar results were obtained for the combinatorial treatment of a cholangiocarcinoma cell line [39]. The HCC cell lines analyzed so far expressed the CSC marker EpCAM (Huh7, HepG2 and Hep3B) [40]. The impact of targeting HCC cells expressing the CSC marker CD90 with AKT and mTOR inhibitors remains unverified. In this study, we investigated the sensitivity of the EpCAM^+^ HCC cell line Huh7 and the CD90^+^ HCC related cell line SK-HEP1 as well as EpCAM^+^ primary patient-derived HCC cells towards the dual treatment with AKT and mTOR inhibitors (MK2206 and RAD001) in vitro and in an orthotopic xenotransplantation mouse model in vivo.

## 2. Material and Methods

### 2.1. Establishment of HCC1 Cell Line

Primary human HCC samples (virus hepatitis negative) were received directly after surgery from the University Medical Center Hamburg-Eppendorf in PBS. Tumor tissue was cut in small pieces and suspended in the Tumor Dissociation Kit enzyme mix (Miltenyi Biotec, Bergisch Gladbach, Germany). The tissue suspensions were disintegrated by three repetitions of GentleMACS (Miltenyi Biotec, Bergisch Gladbach) and incubations for 30 min rotating at 37 °C and 5% CO_2_. Large cell aggregates were removed by filtration through a 70 μm filter. Erythrocytes were removed by incubation with erythrocyte lysis buffer. Cell suspension was concentrated to 5 × 10^5^ cells/10 μL for injection into mice. The HCC1 cell line was established after two rounds of orthotopic transplantation in mice.

### 2.2. Cell Culture

The HCC cell lines Hep3B, Huh7 and HCC-related cell line SK-HEP1 were kindly provided by Hans Will from the Heinrich-Pette-Institute, Hamburg, Germany. HCC1 is a new HCC cell line established from a xenotransplant of primary tumor tissue from an HCC patient in NSG mice. Hep3B and HCC1 cells were maintained in DMEM + 10% FCS and 1% penicillin and streptomycin at 37 °C and 5% CO_2_. SK-HEP1 cells were maintained in RPMI1640 + 20% FCS and 1% penicillin and streptomycin at 37 °C and 5% CO_2_. The cells were passaged twice a week using trypsin/EDTA solution, washed and resuspended in the respective cell culture medium. 

### 2.3. Lentiviral Transduction of Cells

Cells used in the xenograft mouse models were transduced with LeGO-iG2-Puro+fLuc2 vector encoding GFP and firefly luciferase using stable lentiviral transduction as described previously [41]. In brief, HEK293T cells were transfected with the respective vector using Lipofectamine 3000 to enhance the plasmid uptake. Afterwards, the media supernatants containing lentiviral particles were collected for three days. Next, target cells were incubated with the supernatants previously harvested and selected using the appropriate antibiotic. Cytotoxicity assays of puromycin were performed prior to selection. Genetically modified cell lines were selected in puromycin for at least 14 days.

### 2.4. RGB Marking and Clonal Outgrowth Assay

RGB marking of Huh7 cells was performed as previously described in detail [42,43]. For the transduction of Huh7 cells, 200,000 cells were plated per well of a 6-well cell culture plate in 1 mL DMEM. RGB marking was carried out using three LeGO vectors pseudotyped with VSV-G, LeGO-Cer2-Puro+ (Cerulean, cyan-blue), LeGO-V2-Puro+ (Venus, green) and LeGO-C2-Puro+ (mCherry, red). Each of the lentiviral vectors encoded the named fluorescent protein of one of the basic colors—red, green or blue—linked to a puromycin resistance by a 2A sequence and expressed under control of a SFFV promoter [44,45]. The titers of the vector preparations were 2.6 × 10^7^–6.4 × 10^7^/mL, as titrated on HEK293T cells and an MOI (multiplicity of infection, functional virus particles per cell) of 1, relative to the number of HEK293T cells used for titration, was added to the Huh7 cells. The process resulted in transduction rates of 45–63% per color and a total transduction rate of 84%. RGB-marked cells were then selected by addition of puromycin (1 μg/mL), thereby removing the 16% untransduced cells. Afterwards, cells were cultivated in culture flasks in their standard cell culture medium (DMEM + 10% FCS and 1% penicillin and streptomycin). For the clonal outgrowth assay, single treatment with either mTOR inhibitor RAD001 (0.2 µM), AKT inhibitor MK2206 (2 µM) or the combination of both inhibitors (RAD001 0.2 µM/MK2206 2 µM) was performed. DMSO was used as control. Cells were maintained at 37 °C and 5% CO_2_. RAD001 was provided by Novartis AG (Basel, Switzerland). MK2206 was obtained from Selleck Chemicals (Houston, TX, USA). The cells were passaged twice a week using trypsin/EDTA solution, washed, and resuspended in the respective cell culture medium containing the inhibitors or DMSO control. Composition of RGB-marked cells was assessed by flow cytometry (FC) using the LSR Fortessa (Becton, Dickinson and Company, Franklin Lakes, NJ, USA) in a weekly schedule for a total of 3 weeks and afterwards in a monthly schedule (up to 6 six months). Prior to flow cytometry, the cells were filtered, thoroughly washed and resuspended in PBS. Non-RGB-marked Huh7-cells were used as negative control. Pictures were taken with an Olympus IX81 microscope with 10× objective and ColorView-II camera. Overlays were generated in Olympus CellP software from pictures taken in the three RGB channels with the following exposure times: blue 500 ms, red 1000 ms and green 1000 ms.

### 2.5. Western Blot Analysis and Densitometric Quantification

Western blotting was performed as previously described [41]. In brief, cell lysates were prepared in NP40 buffer and separated using SDS gel electrophoresis. Afterwards, the proteins were transferred onto a nitrocellulose membrane and incubated with specific primary antibodies against mTOR (#2983, Cell Signaling Technology, Danvers, MA, USA), panAKT (#4685, Cell Signaling Technology), pAKT S473 (#4060, Cell Signaling Technology), S6 (#2217, Cell Signaling Technology), pS6 S240/S244 (#5364, Cell Signaling Technology), pGSK3ß S9 (#9336, Cell Signaling Technology) or HSC70 (#sc-7298, Santa Cruz Biotechnology, Dallas, TX, USA). Afterwards, the membrane was incubated with the appropriate secondary antibodies against anti-mouse-IgG (#7076, Cell Signaling Technology) or anti-rabbit-IgG (#7074, Cell Signaling Technology). The protein expression was analyzed using the LAS-3000 or LAS-4000 Imager from Fuji (Raytest, Straubenhardt, Germany). Densitometric quantification was carried out using AIDA Image Analyser Software Version 3 (Elysia-raytest GmbH, Straubenhardt, Germany). 

### 2.6. Flow Cytometry (FC) Analysis

FC staining was carried out as described by the supplier of the used antibodies. The cells were counted, centrifuged at 300× *g* for 5 min and the supernatant aspirated completely. An amount of 1 × 10^6^ cells was diluted in 98 µL of “MACS buffer” (PBS + 0.5% (*w*/*v*) bovine serum albumin + 2 mM EDTA) and mixed with 2 µL of staining antibody. The samples were incubated in the dark at 4 °C for 10 min. Thereafter, cells were washed with 1 mL of MACS buffer and centrifuged at 300× *g* for 5 min. The supernatant was aspirated completely, and the cells were resuspended in 100 µL of MACS buffer. The samples were stored on ice and in the dark. FC analysis was carried out using the Cytoflex flow cytometer and analyzed with the CytExpert software (both Beckman and Coulter, Brea, CA, USA).

### 2.7. Two-Dimensional Proliferation Assay

An amount of 3000 cells per well was seeded in 100 µL medium into a 96-well flat bottom plate and cultivated at 37 °C and 5% CO_2_. After 24 h, MK2206, RAD001 or the combination of both drugs and vehicle substance DMSO as control were resuspended in medium and added in a total volume of 100 µL. RAD001 was provided by Novartis AG (Basel, Switzerland). MK2206 was obtained from Selleck Chemicals (Houston, TX, USA). The confluence development was analyzed via IncuCyte ZOOM Live Cell Analysis System (Sartorius, Göttingen, Germany) by phase-contrast pictures (4 per well) every 2 h until the cells reached the stationary phase. 

### 2.8. BrdU Assay

An amount of 1 × 10^4^ cells was seeded in 50 μL of DMEM (+10% FCS and 1% penicillin/streptomycin) in 96-well plates and cultivated at 37 °C and 5% CO_2_. After 24 h, AKT inhibitor MK2206 and mTOR inhibitor RAD001, the combination of both drugs or solvent DMSO were added in ascending concentrations. RAD001 was provided by Novartis AG (Basel, Switzerland). MK2206 was obtained from Selleck Chemicals (Houston, TX, USA). After 72 h of inhibitor treatment, BrdU labeling solution was added according to the manufacturer’s instructions and incorporated for 16 h. The absorbance was measured at 370 nm every 5 min for 30 min using a microplate reader (Tecan, Männedorf, Switzerland). 

### 2.9. Three-Dimensional (Tumoroid) Growth Assay

An amount of 3000 cells per well was seeded into a round bottom 96-well ultralow attachment plate (Corning Inc., Corning, NY, USA) in 50 µL sterile filtered medium. The plates were centrifuged for 5 min at 900× *g*. The cells were then cultivated at 37 °C and 5% CO_2_ and controlled every 24 h. After tumoroids were formed, MK2206, RAD001 or the combination of both drugs and vehicle substance DMSO as control were resuspended in medium and added in a total volume of 50 µL. RAD001 was provided by Novartis AG (Basel, Switzerland). MK2206 was obtained from Selleck Chemicals (Houston, TX, USA). Tumoroids where analyzed every 48 h using light microscopy by measuring the diameter with the AxioVision Microscope Software (Carl Zeiss, Oberkochen, Germany).

### 2.10. Orthotopic Xenograft Mouse Model

Immunodeficient NOD-SCID-IL2Rgamma (−/−) (NSG) mice were subjected to tumor injection. For the injection, cell suspension was concentrated to 1 × 10^6^ cells/10 μL and resuspended in 10 μL of Matrigel. The procedure of tumor cells injection in the left lateral liver lobe for each mouse was performed under general anesthesia using isoflurane inhalation. Through a small midline laparotomy, the left liver lobe was gently mobilized out of the peritoneal cavity. A total of 1 × 10^6^ cells, prepared as stated above, was inoculated into the subcapsular parenchyma through a 31-gauge needle. To prevent tumor cell leakage as well as bleeding from the injection site, a 2 × 2 mm absorbable hemostatic patch (Tabotamp, Johnson & Johnson Medical, Ethicon, Neuchâtel, Switzerland), which is an oxidized regenerated cellulose product, was attached on top of the site of injection for a few minutes after withdrawal of the needle. The liver lobe was returned to the peritoneal cavity. The abdominal wall was closed in layers by continuous manner using absorbable synthetic braided suture (6/0-Vicryl) for the abdominal fascia and in an interrupted manner using synthetic, monofilament, nonabsorbable polypropylene suture (6/0-Prolene) for the skin. General follow up was performed on daily basis by weight measurements and monitoring of mice condition and activities.

Monitoring of tumor growth started one week after implantation and was assessed using bioluminescence after intraperitoneal injection of D-luciferin (30 mg/mL). After 10 min, mice were anesthetized and imaged with an IVIS-200 imaging system (PerkinElmer, Waltham, MA, USA). Post-processing and quantification of the emitted photons were performed using the Living Image software (PerkinElmer, Waltham, MA, USA). A three-dimensional (3D) image by Living Image software was generated to adequately assess successful orthotopic tumor engraftment in the liver. For each mouse, a circular region of interest (ROI) encompassing the primary tumor was selected, and the luminescence in each ROI was quantified. Tumor volume was calculated ex vivo by caliper measurement using the following formula [46]:

$$\frac{\mathrm{longest}\;\mathrm{tumor}\;\mathrm{diameter}*{\left(\mathrm{shortest}\;\mathrm{tumor}\;\mathrm{diameter}\right)}^{2}}{2}$$

Treatment with AKT and mTOR inhibitors was started on the same day of tumor detection by bioluminescence. The control group was treated with placebo while the study group was treated with RAD001 and MK2206. RAD001, formulated as a microemulsion, was dosed at 1 mg/kg body weight and administered on a daily basis (Monday–Friday). MK-2206 was formulated in a 30% (*w*/*v*) Captisol solution and administered 3 days/week, dosed at 120 mg/kg. A placebo microemulsion (provided by Novartis) of a 30% (*w*/*v*) Captisol solution served as placebo. The compounds were mixed immediately before being administered orally by gavage in a total volume of 100 μL. Scheduled treatment duration was 21 days before euthanasia. Any mouse who met euthanasia criteria, which were (i) tumor growth reaches 15 mm, (ii) poor general condition and/or (iii) weight loss > 20%, were euthanized immediately. Xenograft tumors were harvested at necropsy, and a portion of each tumor was fixed with 10% formalin for pathology assessment and snap frozen on liquid nitrogen for Western blotting.

### 2.11. Kinase Activity Profiling

For functional kinome profiling a PamStation^®^12 (located at the UCCH Kinomics Core Facility, Hamburg) was used as described previously [47]. To analyze serine/threonine (STK) and tyrosine kinases (PTK) specific STK and PTK PamChip^®^ were used according to the manufacturer’s instructions (PamGene International, ’s-Hertogenbosch, The Netherlands) as well as M-PER Mammalian Extraction Buffer containing Halt Phosphatase Inhibitor and EDTA-free Halt Protease Inhibitor Cocktail (1:100 each; Pierce, Waltham, MA, USA). Protein quantification was performed with the bicinchoninic acid assay according to the manufacturer’s instructions (Sigma-Aldrich, St. Louis, MO, USA). Per array, 1 µg (STK) or 5 µg (PTK) of protein was applied, technical triplicates were analyzed and sequence-specific peptide phosphorylation was detected using a CCD camera and the Evolve software (PamGene International). After quality control, the final signal intensities were log2-transformed and were used for further data analysis via the BioNavigator software version 5.1 (PamGene International). Unless otherwise stated, data are expressed as the average signal intensity (± standard deviation) of the 144 (STK)/196 (PTK) peptide spots based on end levels of the phosphorylation curve. Significant differences were determined using one-way ANOVA comparisons and Dunnett’s test.

### 2.12. Statistical Analysis

Statistical analysis was carried out using GraphPad Prism version 9.0 (GraphPad Inc., San Diego, CA, USA). A *p* value < 0.05 was considered as statistically significant. All analysis of more than two groups was performed by one-way ANOVA with Tukey’s or Dunnett’s post-test, where applicable. Analysis of two groups was performed by an unpaired, two-sided *t*-test. 

### 2.13. Calculation of Combinatory Indices

Drug interactions of MK2206 and RAD001 were analyzed based on the median effect method of Chou and Talalay [48,49]. The CompuSyn software (Paramus, NJ, USA) was used to calculate the combination index (CI). CI values were encoded as following: Very strong synergism CI < 0.1; strong synergism CI 0.1–0.3; synergism CI 0.3–0.7; moderate synergism 0.7–0.85; slight synergism CI 0.85–0.90; nearly additive CI 0.9–1.10; CI values > 1.1 antagonism.

## 3. Results

### 3.1. Establishment of an EpCAM^+^/CD90−HCC Patient-Derived Xenotransplant (HCC1)

**Figure 1:**
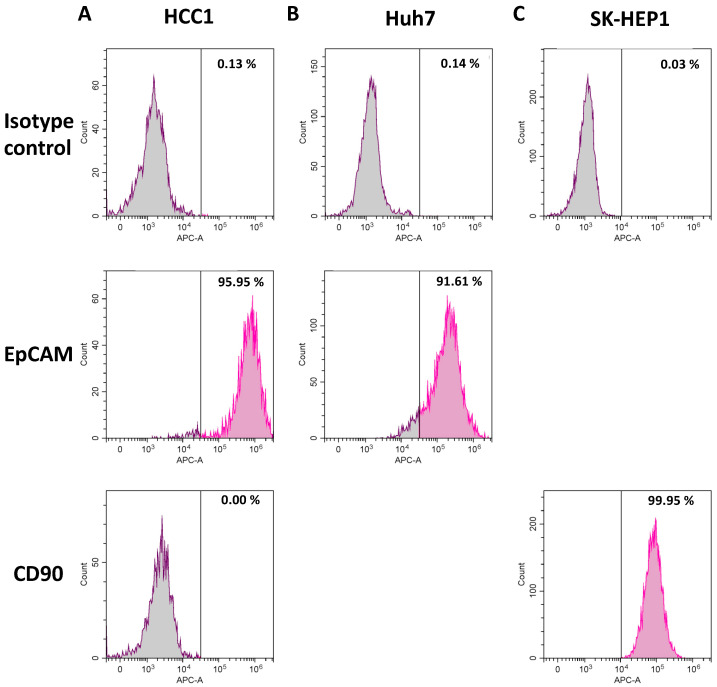
HCC1 cells highly express EpCAM and are negative for CD90. Newly established HCC1 cells (**A**) were analyzed by flow cytometry with APC-labeled antibodies for expression of EpCAM and CD90. The cell lines Huh7 (**B**) and SK-HEP1 (**C**) were used as positive controls for EpCAM and CD90, respectively. APC-labeled isotype control antibodies were used as negative control.

Previously, we demonstrated that the combinatorial treatment of human HCC cell lines—i.e., Huh7, HepG2 and Hep3B—with AKT and mTOR inhibitors led to synergistic effects on cell growth in vitro and in vivo [38]. To further analyze the effects of these inhibitors on primary HCC cells, we collected fresh tumor tissues from HCC patients. Single cells were prepared from HCC tumor pieces by using a gentleMACS, and 1 × 10^6^ cells of each tumor was injected orthotopically in the liver of NSG mice. One out of ten patient-derived HCC cells was able to form a xenograft tumor in the liver after transplantation in mice. In vitro culture of this HCC PDX led to a permanently growing cell line, which we named HCC1. To prove the establishment of a new HCC cell line, an STR profiling was performed, which did not show an identity of HCC1 cells with any cell line in the data bank (Multiplexion Report ID #2983, Heidelberg, Germany). The newly established HCC1 cells showed a strong expression of EpCAM (96%) but no expression of CD90 (0%) (Figure 1).

### 3.2. High Sensitivity of EpCAM^+^/CD90−HCC1 PDX Cells for AKT and mTOR Inhibitors

**Figure 2:**
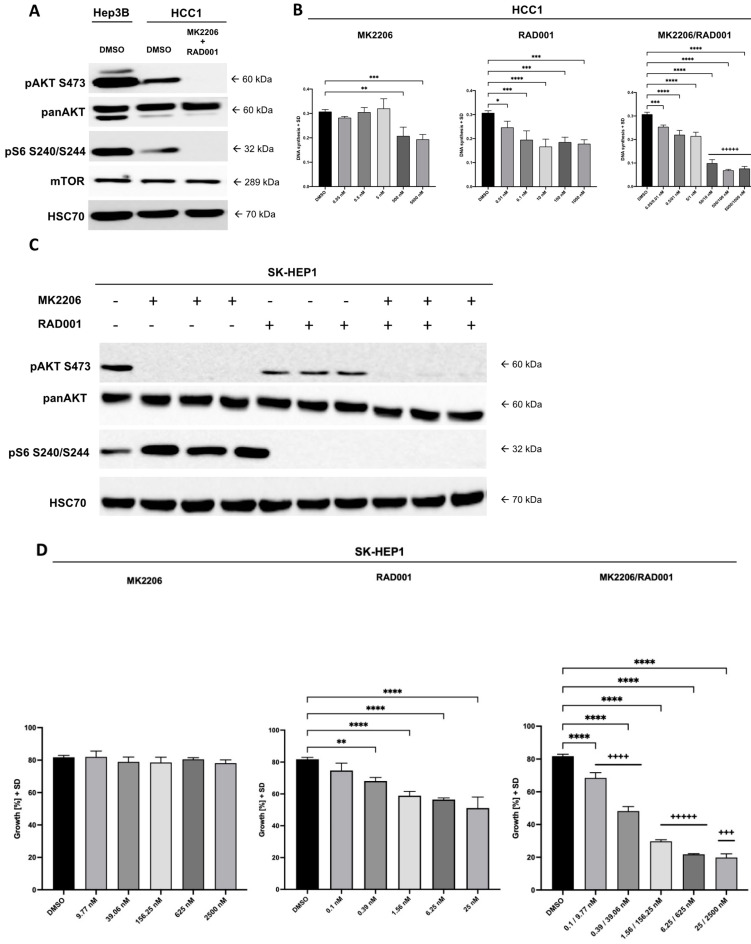
Effect of treatment with AKT and mTOR inhibitors on HCC1 and SK-HEP1 cell growth. (**A**) HCC1 cells were incubated with 5000 nM MK2206 and 1000 nM RAD001 or DMSO control for 14 h and analyzed for expression of phospho-AKT S473, panAKT, phospho-S6 and mTOR by Western blotting. Hep3B cells served as positive control. Uncropped Western blots can be found in Supplementary Figure S8. (**B**) HCC1 cells were incubated for 48 h with the indicated concentrations of MK2206, RAD001 or with both inhibitors. Thereafter, BrdU was added to the culture for 16 h, and DNA synthesis was measured with HRP conjugated anti-BrdU antibodies. The mean values with standard deviation are shown. Combinatory indices were calculated according to the Chou and Talalay method and encoded as described below. (**C**) SK-HEP1 cells were subjected to Western blot analysis to test the susceptibility towards AKT and mTOR inhibition using 5000 nM MK2206, 1000 nM RAD001, the combination of both inhibitors or DMSO control. In total, cells were incubated for 48 h with the respective inhibitor or DMSO control. Uncropped Western blots can be found in Supplementary Figure S8. (**D**) The effect of AKT and mTOR inhibition using MK2206 and RAD001 or the combination of both drugs on SK-HEP1 cells was analyzed in ascending doses using the IncuCyte Zoom live cell imaging system. The confluency of the cells was measured every 2 h. Combinatory indices were calculated according to the Chou and Talalay method and encoded as following: +++++, very strong synergism CI < 0.1; ++++, strong synergism CI 0.1–0.3; +++, synergism CI 0.3–0.7. The *p* values were calculated using one-way ANOVA with Dunnett’s multiple comparisons test (* *p* ≤ 0.05; ** *p* ≤ 0.01; *** *p* ≤ 0.001; **** *p* ≤ 0.0001).

The PI3K–AKT pathway is frequently activated in HCC. Indeed, we were able to observe a constitutive activation of AKT and mTOR detected by phosphorylation of AKT at S473 and the phosphorylation of the mTOR downstream substrate pS6 in HCC1 cells (Figure 2A). Treatment with MK2206 and RAD001 resulted in the complete inhibition of phosphorylation of AKT and S6 (Figure 2A). Effects of treatment with AKT and mTOR inhibitors—i.e., MK2206 and RAD001—on the proliferation of HCC1 cells revealed pronounced sensitivity towards both inhibitors, which was higher for RAD001 (IC_50_ 0.02 nM) than for MK2206 (IC_50_ 139 nM) (Figure 2B). Calculation of combinatory indices as described by Chou and Talalay [48] showed very strong synergistic effects (CI < 0.1) starting at concentrations of 50 nM/10 nM of MK2206 and RAD001, respectively (Figure 2B). 

In order to analyze whether HCC cells that express only the CSC marker CD90 are sensitive for AKT and mTOR inhibitors, we used the HCC-related cell line SK-HEP1 from ascitic fluid that highly expresses CD90 but no other CSC markers, i.e., EpCAM, AFP or CD133 [26]. A strong constitutive activation of the PI3K/AKT/mTOR pathways was confirmed in SK-HEP1 cells (Figure 2C). Similar to HCC1 cells, the SK-HEP1 cells showed high susceptibility towards AKT and mTOR inhibition in vitro, as demonstrated by Western blot analysis (Figure 2C). However, despite the promising suppression of the pAKT expression, the cell line showed an almost complete resistance against AKT inhibition in vitro while retaining the sensitivity for mTOR inhibition (IC_50_: 3.5 nM) (Figure 2D). Dual treatment with AKT and mTOR inhibitors was able to further inhibit SK-HEP1 cell growth compared with single-drug treatment, especially in the intermediate concentrations with very strong to strong synergistic effects (Figure 2D). 

### 3.3. Orthotopic Xenotransplants of HCC1 Cells in NSG Mice Become Resistant against AKT/mTOR Inhibitor Treatment

Recent data have demonstrated many differences between tumor cells that are cultivated in a two-dimensional (2D) culture in comparison with a three-dimensional (3D) culture as tumoroid that is more similar to the tumor growth in vivo. Therefore, we analyzed and compared the data obtained in 2D culture as described above with the same HCC cells grown as tumoroids. 

**Figure 3:**
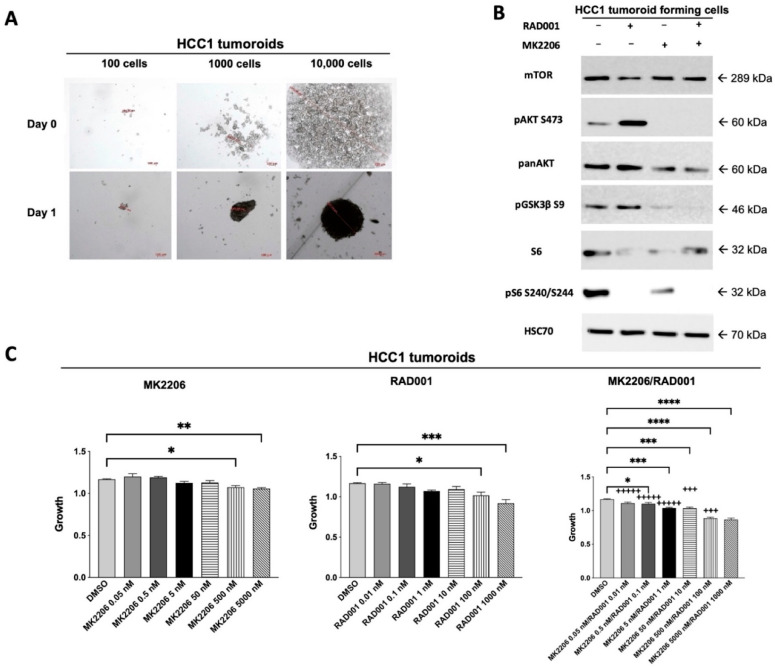

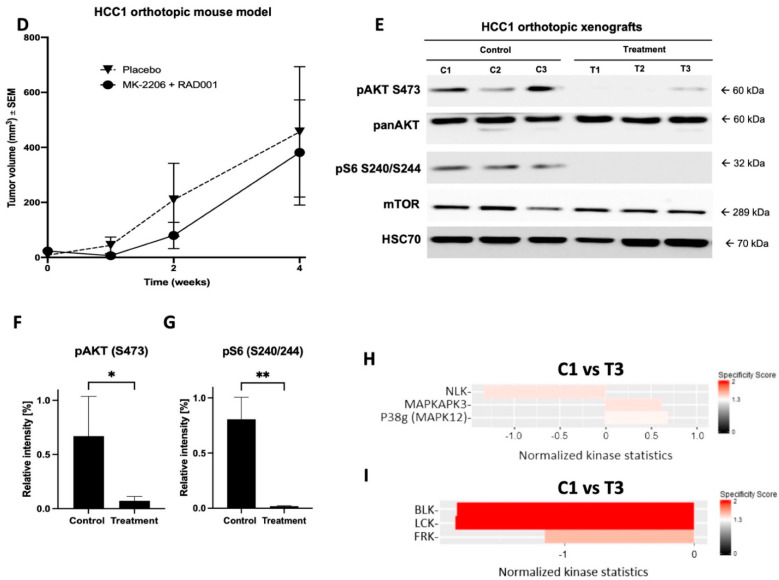
HCC1 xenotransplants become resistant to AKT/mTOR inhibition and show a regulation of distinct serine /threonine and tyrosine kinases. (**A**) HCC1 cells were seeded in ultralow attachment plates, and formation of a tumoroid was observed after one day. (**B**) Tumoroid-forming HCC1 cells were subjected to Western blot analysis after incubation with either MK2206 (5000 nM), RAD001 (1000 nM) or the combination of both inhibitors (MK2206/RAD001 5000 nM/1000 nM) for 24 h. The HCC1 tumoroid-forming cells were sensitive towards AKT and mTOR inhibition as shown by a strong reduction in pAKT (S473) and pS6 (S240/S244) after treatment. Uncropped Western blots can be found in Supplementary Figure S8. (**C**) HCC1 cells were again seeded in ultralow attachment plates and incubated with AKT inhibitor MK2206, mTOR inhibitor RAD001, the combination of both inhibitors or solvent DMSO using the indicated concentrations for a total of 4 days. Afterwards, tumoroid growth was determined by manual measurement of the tumoroid diameter using light microscopy. CI values were calculated according to the Chou and Talalay method and encoded as following: +++++, very strong synergism CI < 0.1; ++++, strong synergism CI 0.1–0.3; +++, synergism CI 0.3–0.7. The *p* values were calculated using one-way ANOVA with Dunnett’s multiple comparisons test (* *p* ≤ 0.05; ** *p* ≤ 0.01; *** *p* ≤ 0.001; **** *p* ≤ 0.0001). (**D**) HCC1 cells were orthotopically injected into the left lateral liver lobe of NSG mice (*n* = 3 per group). Mice were treated orally either with the combination of AKT inhibitor MK2206 (120 mg/kg) and mTOR inhibitor RAD001 (1 mg/kg) or placebo control for a total of three weeks. (**E**) Western blot analysis of the orthotopic xenograft tumors grown in the left lateral liver lobe of NSG mice. Densitometric quantification of the pAKT (S473) expression (**F**) and pS6 expression (**G**) of the xenograft tumors after treatment with AKT and mTOR inhibitors in vivo as shown in (**E**) revealed that the inhibitors inhibited their respective target. The *p* values were calculated using an unpaired, two-sided T-test (* *p* ≤ 0.05; ** *p* ≤ 0.01). Uncropped Western blots can be found in Figure S8. The first xenograft tumor of the control group (C1) and the third tumor from the treatment group (T3) with detectable pAKT (S473) were subjected to serine/threonine kinase (**H**) and tyrosine kinase (**I**) profiling using PamGene assays with microarrays containing immobilized peptide sequences with 13 amino acids per sequence representing kinase-specific phosphorylation sites. Only significantly up- or downregulated kinases are displayed in (**H**,**I**). The full list of all kinases analyzed can be found in Supplementary Figures S6 and S7.

HCC1 cells formed tumoroids under ultralow attachment conditions within 24 h after seeding (Figure 3A). Again, Western blot analysis of the tumoroid-forming HCC1 cells revealed a strong activation of the PI3K/AKT/mTOR signaling pathway that is susceptible to AKT and mTOR inhibition (Figure 3B). However, the HCC1 tumoroids were less susceptible to AKT and mTOR inhibitors compared with the 2D culture in vitro (Figure 3C). In a next step, we orthotopically injected HCC1 cells into the left liver lobe of NSG mice (Supplementary Figures S1–S5). After one week of tumor growth, we started to treat the mice by oral administration with AKT and mTOR inhibitors—i.e., MK-2206 and RAD001. In accordance with the results from the drug sensitivity testing of 2D cultures and 3D cultures in vitro, we observed an initial reduction in tumor growth after one week of treatment. However, thereafter the tumor started to grow over the next weeks, indicating the development of resistance against the AKT/mTOR inhibitor treatment (Figure 3D). Western blot analysis of the orthotopic xenograft tumors revealed a suppressed pS6 (S240/S244) in all treated samples, indicating that RAD001 still inhibited mTOR/p70S6K signaling in the resistant tumor cells (Figure 3E,F). However, expression of pAKT (S473) was partly restored in one out of three xenograft tumors (T3), indicating an upregulation of AKT phosphorylation at the regulatory serine 473 residue in this HCC1 xenograft despite the treatment with AKT inhibitor (Figure 3F). To identify additional kinases that are regulated during the development of resistance against the AKT/mTOR inhibitor treatment, we subjected protein lysates from this tumor tissue (T3) and from the tumor tissue of an untreated control mouse (C1) to serine/threonine and tyrosine kinase profiling using PamGene micro arrays. Interestingly, in the T3 xenograft tumor with detectable pAKT (S473), the serine/threonine kinase NLK was significantly downregulated, whereas MAPAPK3 and p38 MAPK were significantly upregulated compared with xenograft tumor C1 (Figure 3H). Among the tyrosine kinases, the SRC family kinases BLK and LCK as well as the SRC family-related kinase FRK were significantly downregulated in the pAKT-positive T3 xenograft tumor (Figure 3I). 

### 3.4. Combined AKT and mTOR Inhibition Increases Overall Survival of Mice after Orthotopic Transplantation of CD90^+^ SK-HEP1 Cells

**Figure 4:**
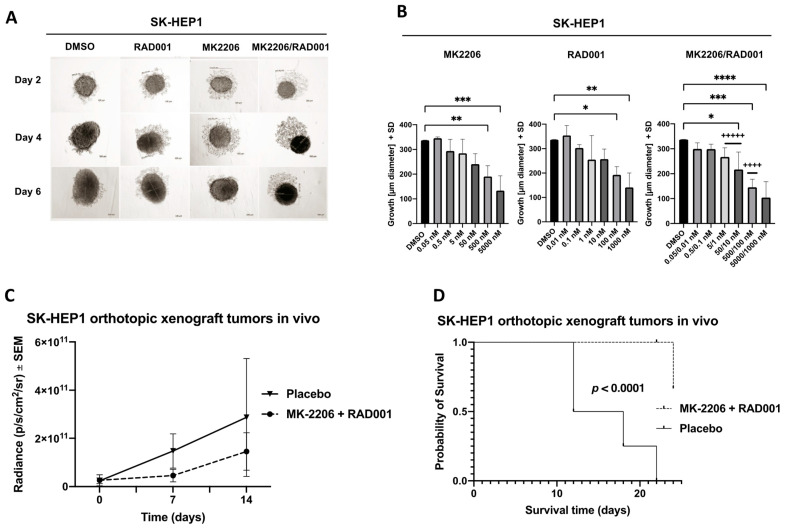

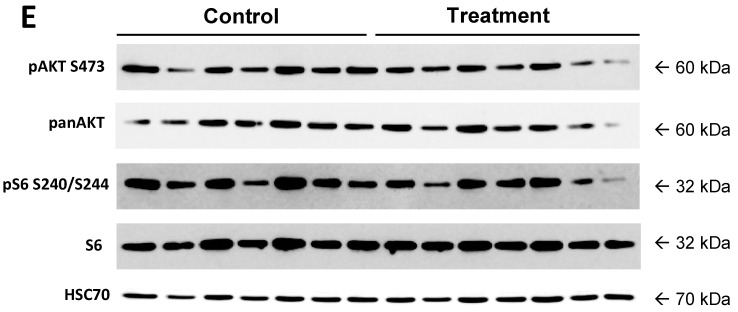
Combined AKT and mTOR inhibition increases overall survival of mice after orthotopic SK-HEP1 tumor formation. (**A**) Representative images of SK-HEP1 cells that were seeded into 96-well ultralow attachment plates and allowed to form tumoroids for two days. Afterwards, tumoroids were treated for 96 h with serial dilutions of AKT inhibitor MK2206 (500 nM shown), mTOR inhibitor RAD001 (100 nM shown), the combination of both inhibitors (500 nM/100 nM shown) or solvent DMSO. (**B**) The tumoroid diameter of the SK-HEP1 tumoroids was calculated using light microscopy 4 days after of AKT and mTOR inhibitor treatment using the indicated concentrations. CI values were calculated according to the Chou and Talalay method and encoded as following: +++++, very strong synergism CI < 0.1; ++++, strong synergism CI 0.1–0.3. The *p* values were calculated using one-way ANOVA with Dunnett’s multiple comparisons test (* *p* ≤ 0.05; ** *p* ≤ 0.01; *** *p* ≤ 0.001; **** *p* ≤ 0.0001). (**C**) SK-HEP1 cells were orthotopically injected into the left lateral liver lobe of NSG mice (*n* = 8 per group). Mice were treated orally either with the combination of AKT inhibitor MK2206 (120 mg/kg) and mTOR inhibitor RAD001 (1 mg/kg) or placebo control for a total of three weeks. The bioluminescence was measured every week. (**D**) Kaplan–Meier curves displaying the overall survival of the mice after orthotopic injection of SK-HEP1 cells and treatment with AKT/mTOR inhibitors or placebo. (**E**) Western blot analysis of the orthotopic xenograft tumors grown in the left lateral liver lobe of NSG mice as shown in (**C**). Uncropped Western blots can be found in Supplementary Figure S8.

In a next step, we analyzed the effects of a combined treatment with AKT and mTOR inhibitors on the CD90^+^ HCC-related cell line SK-HEP1 which successfully formed tumoroids under ultralow attachment conditions (Figure 4A). Treatment with AKT inhibitor MK2206 (IC_50_: 72.2 nM), mTOR inhibitor RAD001 (IC_50_: 22.2 nM) or the combination of both drugs affected tumoroid growth in a concentration-dependent manner with strong to very strong synergistic effects especially in intermediate concentrations (Figure 4B). 

Additionally, we tested the sensitivity of SK-HEP1 xenograft tumors for MK2206 and RAD001 treatment after orthotopic intrahepatic injection of the cells. Treatment with AKT and mTOR inhibitors was able to decrease the tumor burden of mice (Figure 4C) and significantly increase the probability of survival in the treatment group (*p* < 0.0001) (Figure 4D). Western blot analysis of the excised tumors revealed a partial or even complete restoration of pAKT (S473) and pS6 (S240/S244) signaling in all xenograft tumors resistant to AKT and mTOR inhibitors (Figure 4E). 

### 3.5. Reduced Tumor Growth of Orthotopically Transplanted HCC Cells in NSG Mice by AKT and mTOR Inhibitors

**Figure 5:**
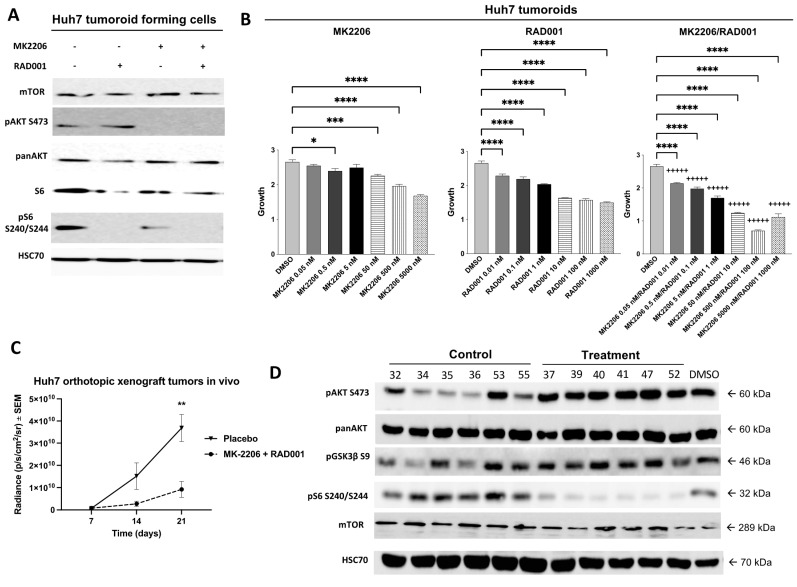

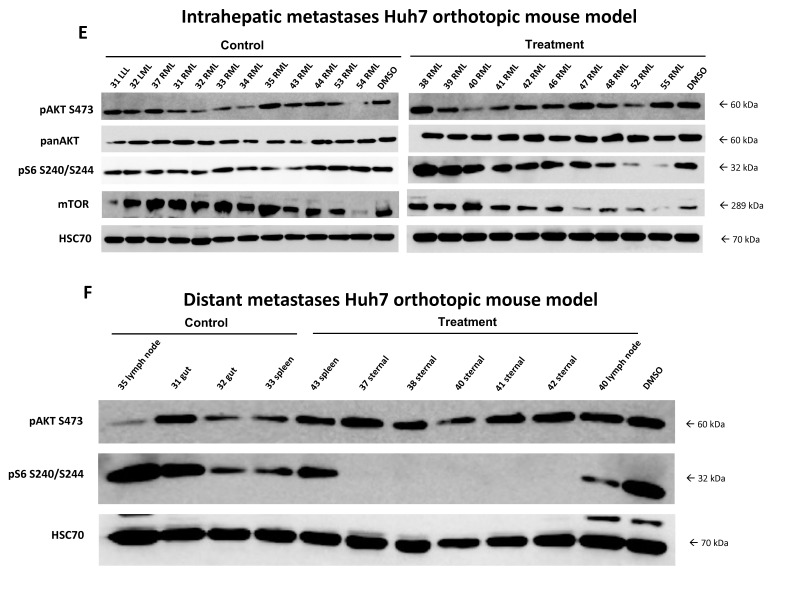

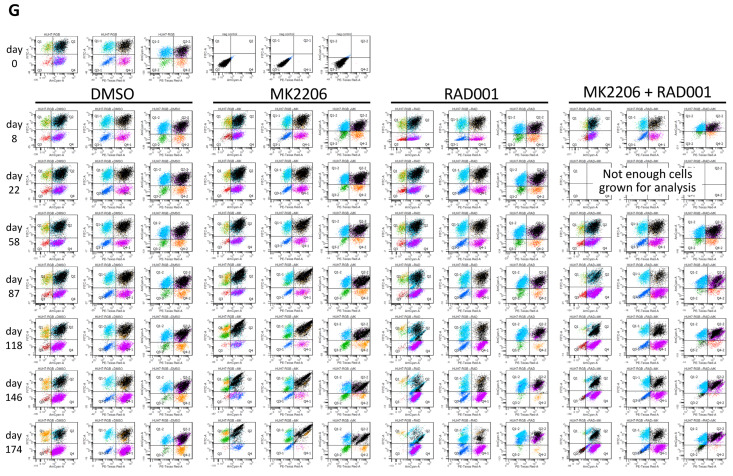

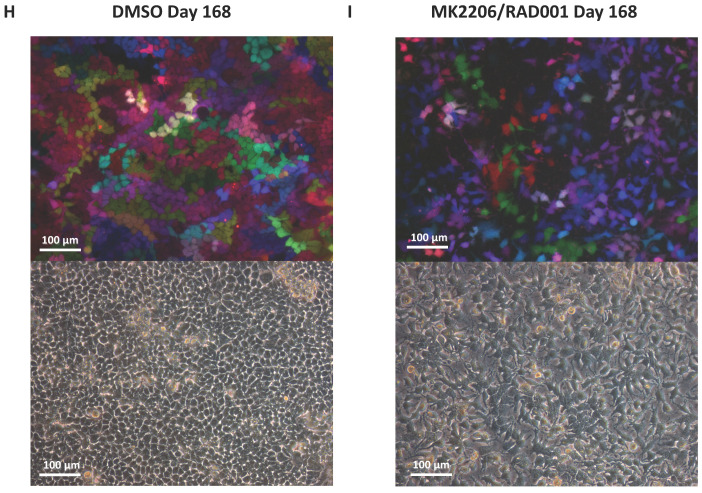
Combined targeting of AKT and mTOR is highly synergistic in vitro but leads to treatment resistance of xenograft tumors in an orthotopic model in vivo. (**A**) Tumoroid-forming Huh7 cells were subjected to Western blot analysis to test the susceptibility of the cells towards AKT and mTOR inhibition. (**B**) Huh7 tumoroids were treated with AKT inhibitor MK2206, mTOR inhibitor RAD001, the combination of both inhibitors or solvent DMSO. CI values were calculated according to the Chou and Talalay method and encoded as following: +++++, very strong synergism CI < 0.1. The *p* values were calculated using one-way ANOVA with Dunnett’s multiple comparisons test (* *p* ≤ 0.05; *** *p* ≤ 0.001; **** *p* ≤ 0.0001). (**C**) Huh7 cells were orthotopically injected into the left lateral liver lobe of NSG mice (*n* = 8 per group, ** *p* ≤ 0.01). Mice were randomized in two groups and treated orally either with the combination of AKT inhibitor MK2206 (120 mg/kg) and mTOR inhibitor RAD001 (1 mg/kg) or placebo control. The bioluminescence was measured every week. Afterwards, Western blot analysis of the orthotopic xenograft tumors grown in the left lateral liver lobe of NSG mice (**D**), the intrahepatic metastases (**E**) and the distant metastasis (**F**) was conducted. (**G**) Scatter plots of RGB-marked Huh7 cells that were exposed continuously for a total duration of approximately 6 months (174 days) to DMSO control, MK2206 single treatment (2 µM), RAD001 single treatment (0.2 µM) or the combination of both drugs (MK2206 2 µM/RAD001 0.2 µM). Representative pictures of RGB-marked Huh7 cells using fluorescence (10×, exposure times: blue 500 ms, red 1000 ms and green 1000 ms) and phase-contrast microscopy (10×) after approximately 6 months (168 days) of continuous exposure to DMSO control (**H**) or combinatorial AKT/mTOR treatment with MK2206/RAD001 (2 µM/0.2 µM) (**I**). Uncropped Western blots can be found in Supplementary Figure S8.

In a next step, we investigated whether the EpCAM^+^ tumoroid-forming Huh7 cells are susceptible to treatment with AKT and mTOR inhibitors. Similar to the HCC1 and SK-HEP1 cell line, a robust inhibition of pAKT (S473) and pS6 (S240/S244) after treatment with MK2206 and RAD001 in vitro was detected (Figure 5A). Very strong synergistic effects could be observed along the entire dose gradient by dual targeting AKT as well as mTOR in Huh7 tumoroids (Figure 5B). We recently demonstrated that combinatorial treatment with AKT and mTOR inhibitors results in reduced tumor growth and increased survival of mice after subcutaneous transplantation of EpCAM^+^ Huh7 cells [38]. 

In order to analyze whether this combinatorial treatment will also reduce the growth of tumors in the liver, we used our orthotopic HCC mouse model. Mice were classified into study and control groups (*n* = 8 per group) for treatment with placebo control or the combination of AKT and mTOR inhibitors for 21 days, which resulted in a significant reduction in tumor growth (*p* = 0.0068) in the treatment group (Figure 5C). However, treatment was not able to improve overall survival (data not shown). Interestingly, Western blot analysis revealed a strong activation of pAKT (S473) in all xenograft tumors (Figure 5D) in most of the intrahepatic metastases (Figure 5E) and all distant metastases (Figure 5F). 

We further analyzed whether the development of resistance against AKT and mTOR inhibitor treatment is a phenomenon that occurs in many cells or in a few cells only. For this purpose, we exposed red-green-blue (RGB)-marked Huh7 cells to treatment with MK2206, RAD001 or the combination of both drugs or solvent DMSO and repeatedly examined the cells for clonal outgrowth by using FC analysis for a total of 6 months (Supplementary Figure S8). Initially, the cells that were exposed to dual AKT/mTOR inhibition showed a strong growth suppression but no distinct clonal outgrowth after 2 months (Figure 5G). Over the course of treatment, first dominant clones were visible after approximately 3 months (87 days) of treatment and continued to grow in more evident resistant clones in the following 3 months. The results demonstrate that a large number of RGB-marked Huh7 cells became resistant against AKT and mTOR inhibitor treatment (Figure 5G). These results were confirmed by fluorescence microscopy that revealed the outgrowth of many different RGB-marked Huh7 cells (Figure 5H,I). Similar results were obtained by analyzing the RGB-marked Huh7 cells during the treatment with either MK2206 or RAD001 alone (Figure 5G). These data indicate that the acquisition of resistance against AKT and mTOR inhibitors occurs quite fast and in a large number of Huh7 cells.

## 4. Discussion

EpCAM and CD90 have been implicated as CSC markers in HCC due to their association with increased tumorigenicity, metastasis and poor prognosis of HCC patients. CD90^+^ CSCs have been purified from HCC cell lines as well as from tissue samples of HCC patients. In cellular metabolism, CD90^+^ CSCs regulate cell-to-cell and cell-to-matrix interaction, apoptosis, adhesion, migration and fibrosis [50]. In HCC cell lines, CD90^+^ CSCs have a tumorigenic capacity when injected into immune-deficient mice [51]. Moreover, CD90 is highly expressed in poorly differentiated HCC and is associated with poor prognosis [25,27,52,53]. Additionally, the surface marker EpCAM was identified in the past to play a crucial role in HCC, as it is associated with shorter overall survival rates and higher rates of portal vein invasion and is enriched in the invasive border of HCCs [26,54]. However, CSCs are characterized by a high degree of resistance to chemo- and radiotherapy [54,55] that makes it difficult to treat the disease in curative intent and to prevent relapse. Therefore, the importance of novel therapeutic targets in CD90^+^ and EpCAM^+^ CSCs to eradicate HCC is becoming increasingly relevant. Ultimately, new treatment strategies targeting not only tumor bulk mass but also HCC-associated CSCs are of high importance [56]. In this study, we examined the sensitivity of CD90^+^ and EpCAM^+^ cells for AKT and mTOR inhibitors in vitro and in an orthotopic mouse model in vivo. Additionally, from a hepatocellular carcinoma patient, we established a novel HCC cell line, named HCC1, that exhibits a strong EpCAM but no CD90 expression. 

### 4.1. CD90^+^ Cells Derived from Ascitic Fluid of an HCC Patient Develop Resistance against Combined Targeting of AKT/mTOR by Restauration of AKT and mTOR Signaling

CD90^+^ SK-HEP1 cells grown in regular 2D cultures or grown as 3D tumoroids were highly sensitive for the combinatorial treatment with AKT and mTOR inhibitors in vitro. In addition, we observed a slower tumor progression and increased survival after combinatorial treatment of the SK-HEP1 tumors in the orthotopic mouse model in vivo. Nevertheless, all xenotransplanted tumors became resistant to AKT/mTOR treatment. Further analysis revealed a partial or even complete restauration of the initial pAKT and pS6 expression in all treated tumors analyzed. These data indicate that the CD90^+^ SK-HEP1 cells quickly develop a resistance against the AKT/mTOR inhibitor treatment, most likely by restoration of the initial AKT and mTOR signaling. Of note, the origin of the SK-HEP1 cells is controversial, as they were initially classified as hepatocellular adenocarcinoma cells. However, later studies instead indicate an endothelial origin due to their characteristics and the lack of hepatic-specific protein expression [57]. Nevertheless, the SK-HEP1 have been utilized as a model of CD90^+^ HCC CSCs in the past [26].

### 4.2. EpCAM^+^ HCC Cells Resistant against AKT and mTOR Inhibitors Upregulate the p38MAPK Pathway

In the past, a high sensitivity of EpCAM^+^ Huh7 cells was determined in 2D cell culture and in subcutaneous xenograft mouse models [38]. In line with these data, we were able to observe a high sensitivity and very strong synergistic effects along the entire dose gradient of Huh7-derived tumoroids against AKT/mTOR inhibition. In addition to that, we were able to demonstrate that combined targeting of AKT and mTOR significantly decreases tumor burden in our orthotopic xenograft mouse model after transplantation into the left liver lobe. Nevertheless, AKT and mTOR inhibition was not able to prolong overall survival of mice of to prevent local and distant metastasis. Interestingly, molecular characterization of the xenograft tumors revealed a strong restauration of pAKT (S473) while retaining a suppressed pS6 expression due to mTOR inhibition in all treated tumors. The same difference was observed for the distant metastases that also showed a restoration of pAKT but not pS6 expression. A possible explanation for this observation may be that mTOR inhibition can trigger a feedback loop leading to the activation of PI3K and thereby consecutively hyperactivates AKT [36]. A reactivation of mTOR may therefore counteract the activity of the PI3K/AKT pathway and may not have a selective advantage during the development of treatment resistance. Interestingly, in the intrahepatic metastases, we observed activation of both pAKT and pS6 expression in all metastases analyzed, suggesting a specialized role of mTOR/S6 kinase signaling for the growth of therapy-resistant metastatic cells within the liver.

An interesting question remains regarding the cause of the fast development of resistance against the AKT and mTOR inhibitor treatment observed in HCC cells in vitro and in vivo. One possible explanation may be the rapid outgrowth of CSCs that are resistant to AKT/mTOR treatment. Indeed, an increased resistance of cancer cells and CSCs modulated by the AKT axis has been described in the past [58]. Another mechanism of resistance may be the activation of closely related signaling pathways such as the RAS/RAF/MEK/MAPK signaling pathway [59]. Interestingly, we identified an upregulation of the stress-activated mitogen kinase p38 as well as one member of the p38 signaling pathway—i.e., the MAPK activated protein kinase 3 (MAPKAPK3)—in a mouse tumor that developed under AKT/mTOR treatment in comparison with a mouse tumor that developed in an untreated control mouse. This pathway has many functions during stress response and cancer development, but MAPKAPK3 is also able to phosphorylate Beclin 1, thereby positively regulating starvation-induced autophagy [60]. Moreover, a crucial role of activated p38 MAPK pathway has been described in cancer with respect to cellular survival, migration and invasion [61]. Such a physiological mechanism that may contribute to the resistance against AKT/mTOR treatment would be in line with our observation from the clonality studies of RGB-marked cells indicating that large numbers of HCC cells become resistant to AKT/mTOR inhibitor treatment. 

We further analyzed the regulation of tyrosine kinases as putative upstream regulators of the observed restoration of AKT activation in the resistant HCC tumor cells. Src kinases play a crucial role in intracellular signal transduction and contribute to the regulation of proliferation, cellular motility and adhesion as well as differentiation [62]. In the past, Lu et al. reported that activated Src kinases are able to alter the function of the tumor suppressor and negative regulator of the PI3K/AKT/mTOR pathway PTEN by interfering with the membrane-binding capacity of PTEN, leading to increased pAKT expression [63]. Surprisingly, we found a downregulation rather than an upregulation of several Src kinase and Src kinase-related family members—i.e., B-lymphoid tyrosine kinase (BLK), lymphocyte-specific protein tyrosine kinase (LCK) and Fyn-related kinase (FRK)—in the AKT/mTOR-inhibitor-resistant tumor cells. 

## 5. Conclusions

In conclusion, we observed high sensitivity of EpCAM^+^ and CD90^+^ HCC cells for the inhibition of proliferation with AKT and mTOR inhibitors in 2D and 3D models in vitro. However, EpCAM^+^ HCC1 and Huh7 cells as well as CD90^+^ HCC-derived SK-HEP1 cells rapidly developed resistance against the combined treatment with AKT and mTOR inhibitors in orthotopic liver cancer models in mice. The development of resistance was observed in large numbers of distinct MK2206/RAD001-treated Huh7 cells, as shown by RGB marking. Together our data from in vitro and in vivo experiments indicate that the acquisition of resistance against AKT and mTOR inhibitors occurs fast and in a large number of Huh7 cells.

Restoration of pAKT was observed in most tumors during AKT/mTOR therapy, underlining the importance of restored AKT signaling as a resistance mechanism. One possible explanation may be the observed upregulation of the p38 MAPK pathway during resistance development. Further work is required to analyze the molecular mechanisms of the rapid treatment resistance of HCC cells in order to achieve a better treatment of HCC patients by eradicating their CSCs. 

## Data Availability

Data are contained within the article or its Supplementary Materials.

## Supplementary Materials

The following supporting information can be downloaded at: https://www.mdpi.com/article/10.3390/cancers14081882/s1, Figure S1: Implantation of human-derived HCC cells into the left lateral lobe of NSG-mouse liver parenchyma, Figure S2: Abdominal sonography of NSG-mouse to detect liver parenchyma tumor growth and to measure its dimension after orthotopic injection of HCC cells, Figure S3: Bioluminescence screening to early detect tumor engraftment in the liver of NSG-mouse, Figure S4: Harvested NSG-mouse liver showing tumor growth in the liver parenchyma, Figure S5: Histologic assessment of the liver specimens revealed infiltrates of malignant tumor cells without evident lineage. The tumor cells were mostly plump, partially spindled and showed highly pleomorphic nuclei with cleared chromatin and prominent nucleoli. Tumors were unencapsulated and showed a dissecting growth pattern between adjacent hepatocytes. The central aspects of the tumors showed large areas of necrosis. The morphologic findings are in keeping with prior descriptions of SK-HEP1 infiltrates, Figure S6: Complete list of up- or downregulated serine/threonine kinases of the third tumor from the treatment group (T3) in comparison with the first HCC1 xenograft tumor of the control group (C1) after profiling using PamGene assays as described in Figure 3H, Figure S7: Complete list of up- or downregulated tyrosine kinases of the third tumor from the treatment group (T3) in comparison with the first HCC1 xenograft tumor of the control group (C1) after profiling using PamGene assays as described in Figure 3I, Figure S8: Original uncropped Western blots.
