# Supplementary material for: Combined Targeting of AKT and mTOR Inhibits Tumor Formation of EpCAM+ and CD90+ Human Hepatocellular Carcinoma Cells in an Orthotopic Mouse Model
Source: Cancers (Basel). 2022 Apr 8;14(8):1882. doi: 10.3390/cancers14081882 (PMC9024696; doi:10.3390/cancers14081882)
Supplement: Supplementary file 1 [file cancers-14-01882-s001.zip › cancers-1641607-supplementary.pdf]

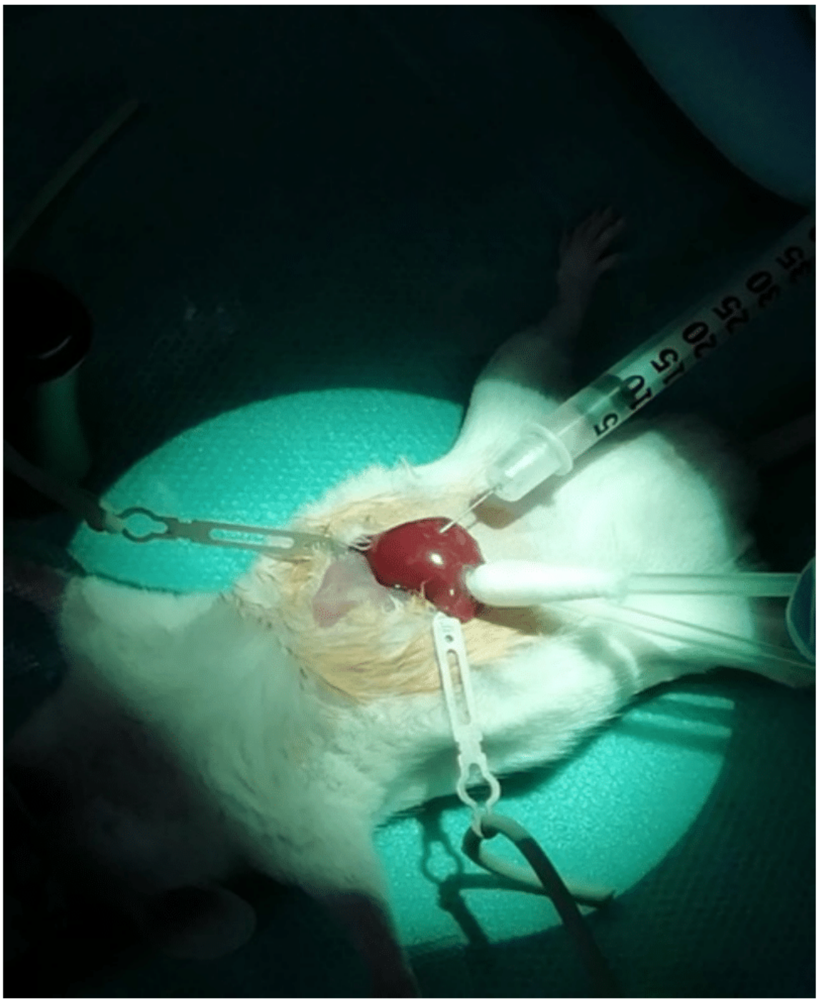

Figure S1. Implantation of human-derived HCC cells into the left lateral lobe of NSG-mouse liver parenchyma

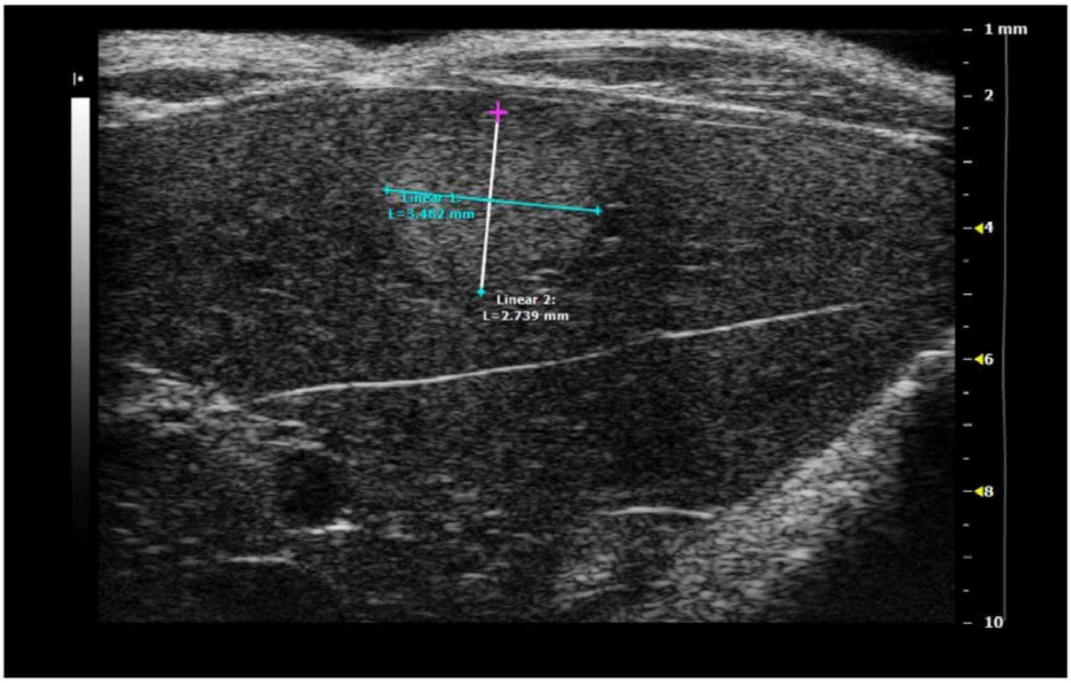

Figure S2. Abdominal sonography of NSG-mouse to detect liver parenchyma tumor growth and to measure its dimension after orthotopic injection of HCC cells

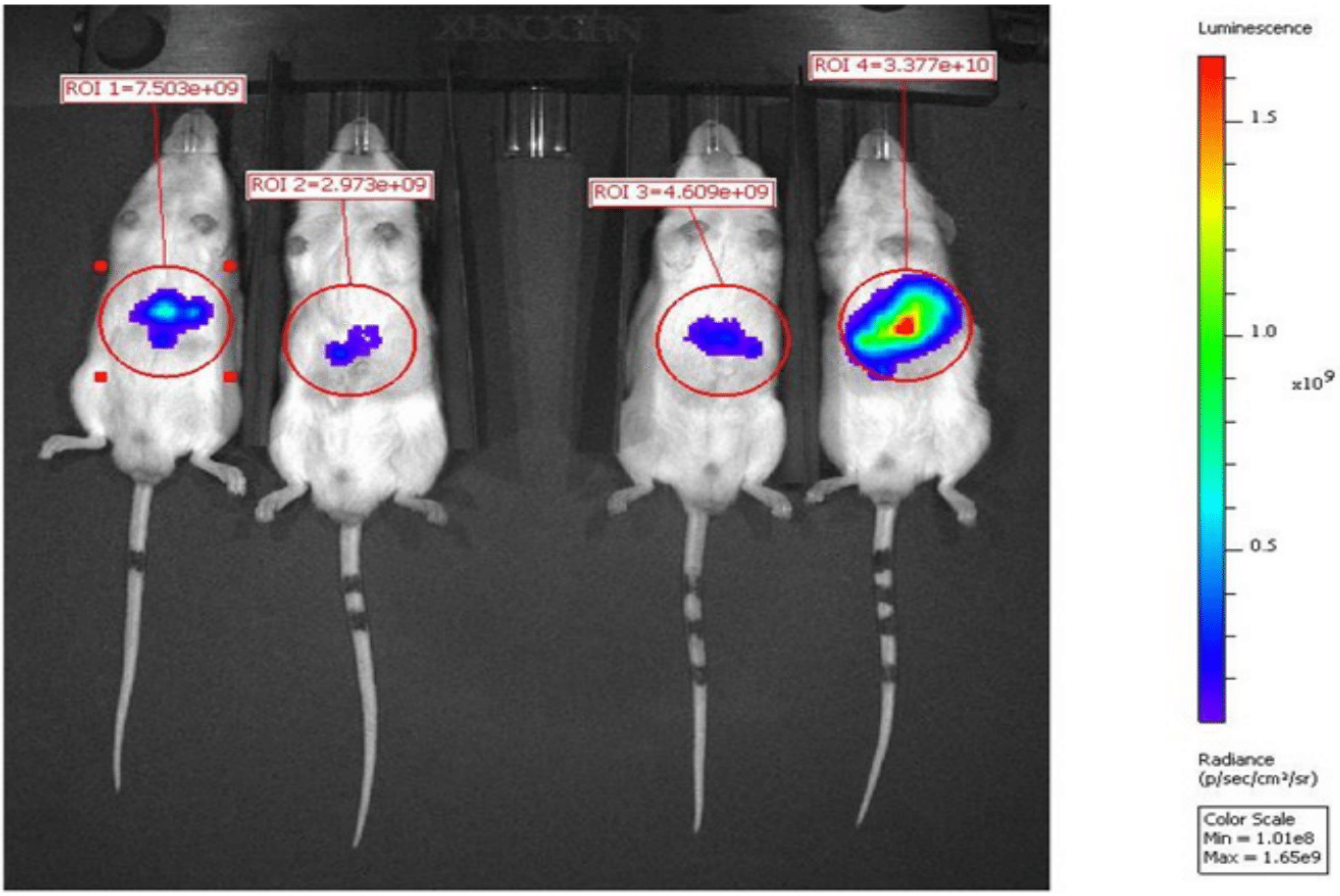

Figure S3. Bioluminescence screening to early detect tumor engraftment in the liver of NSG-mouse

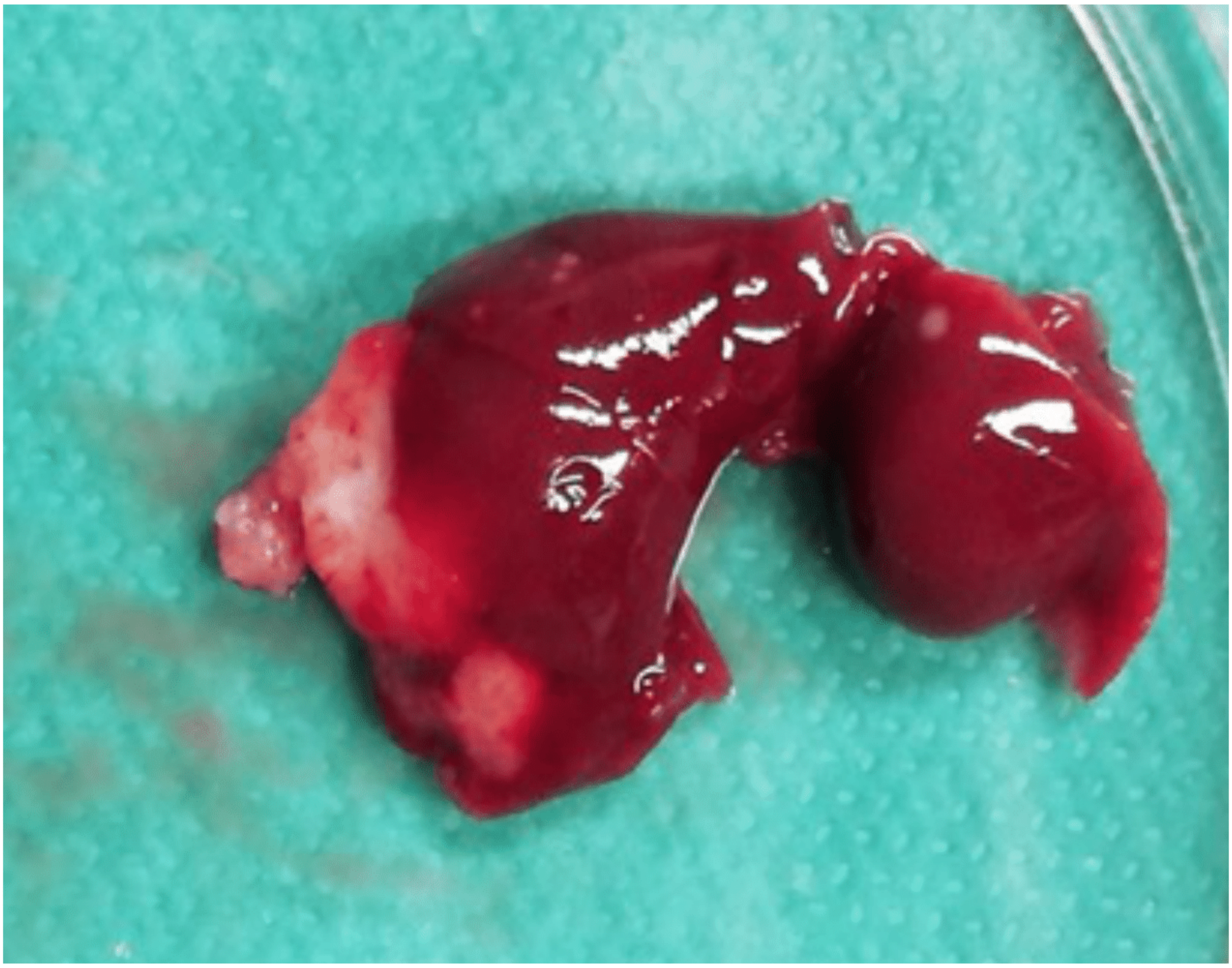

Figure S4. Harvested NSG-mouse liver showing tumor growth in the liver parenchyma

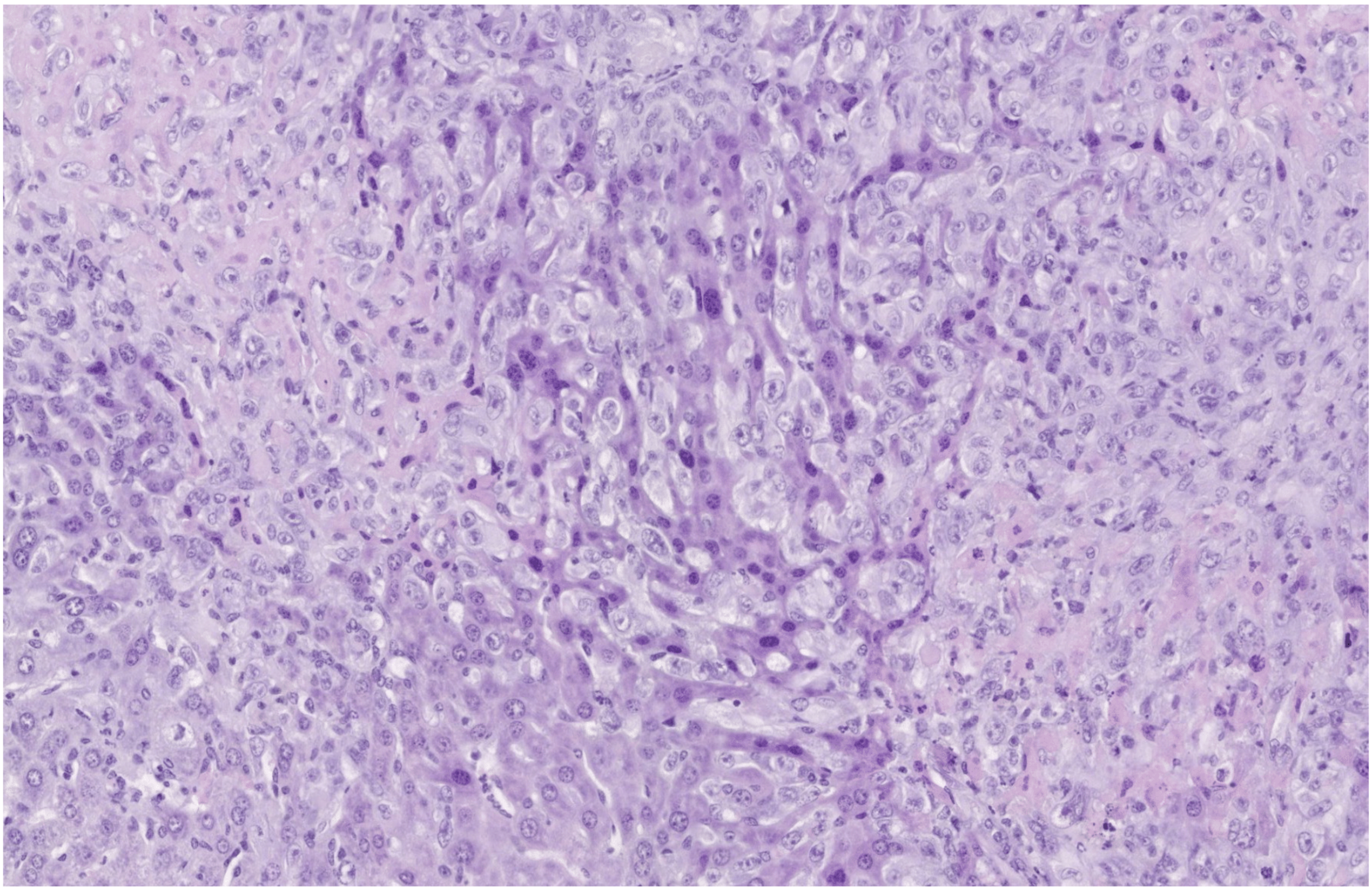

Figure S5. Histologic assessment of the liver specimens revealed infiltrates of malignant tumor cells with-out evident lineage. The tumor cells were mostly plump, partially spindled and showed highly pleomorphic nuclei with cleared chromatin and prominent nucleoli. Tumors were unencapsulated and showed a dissecting growth pattern between adjacent hepatocytes. The central aspects of the tumors showed large areas of necrosis. The morphologic findings are in keeping with prior descriptions of SK-HEP1 infiltrates

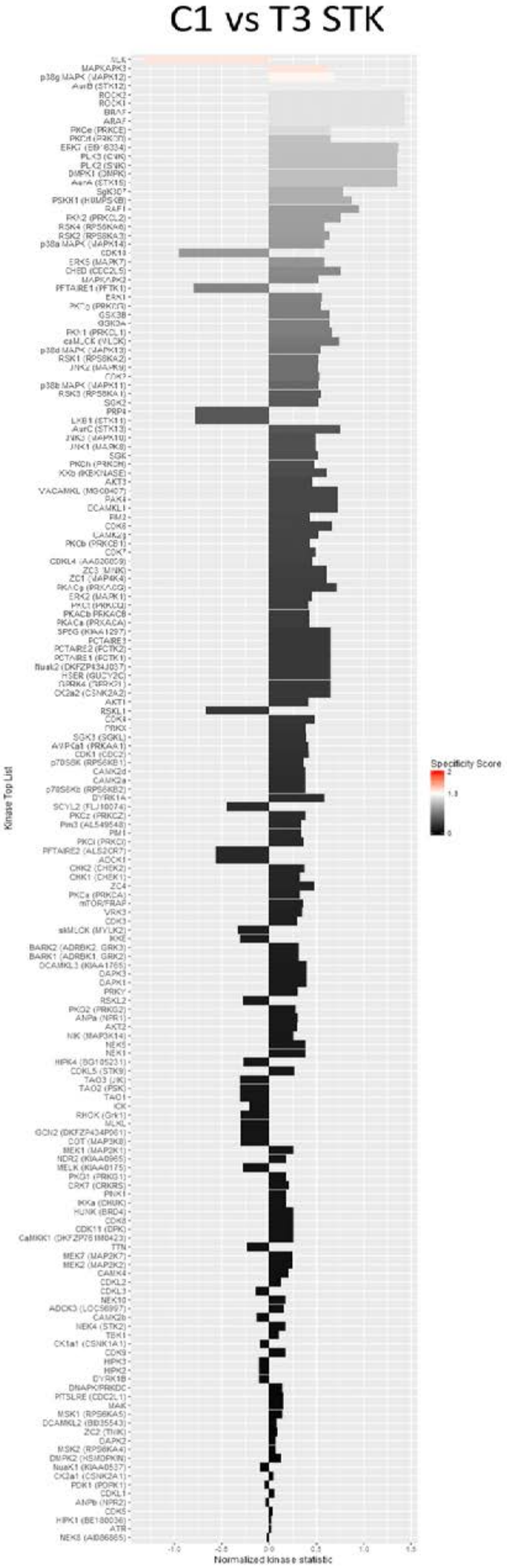

Figure S6. Complete list of up- or downregulated serine/threonine kinases of the third tumor from the treatment group (T3) in comparison with the first HCC-1 xenograft tumor of the control group (C1) after profiling using PamGene assays as described in Figure 3H

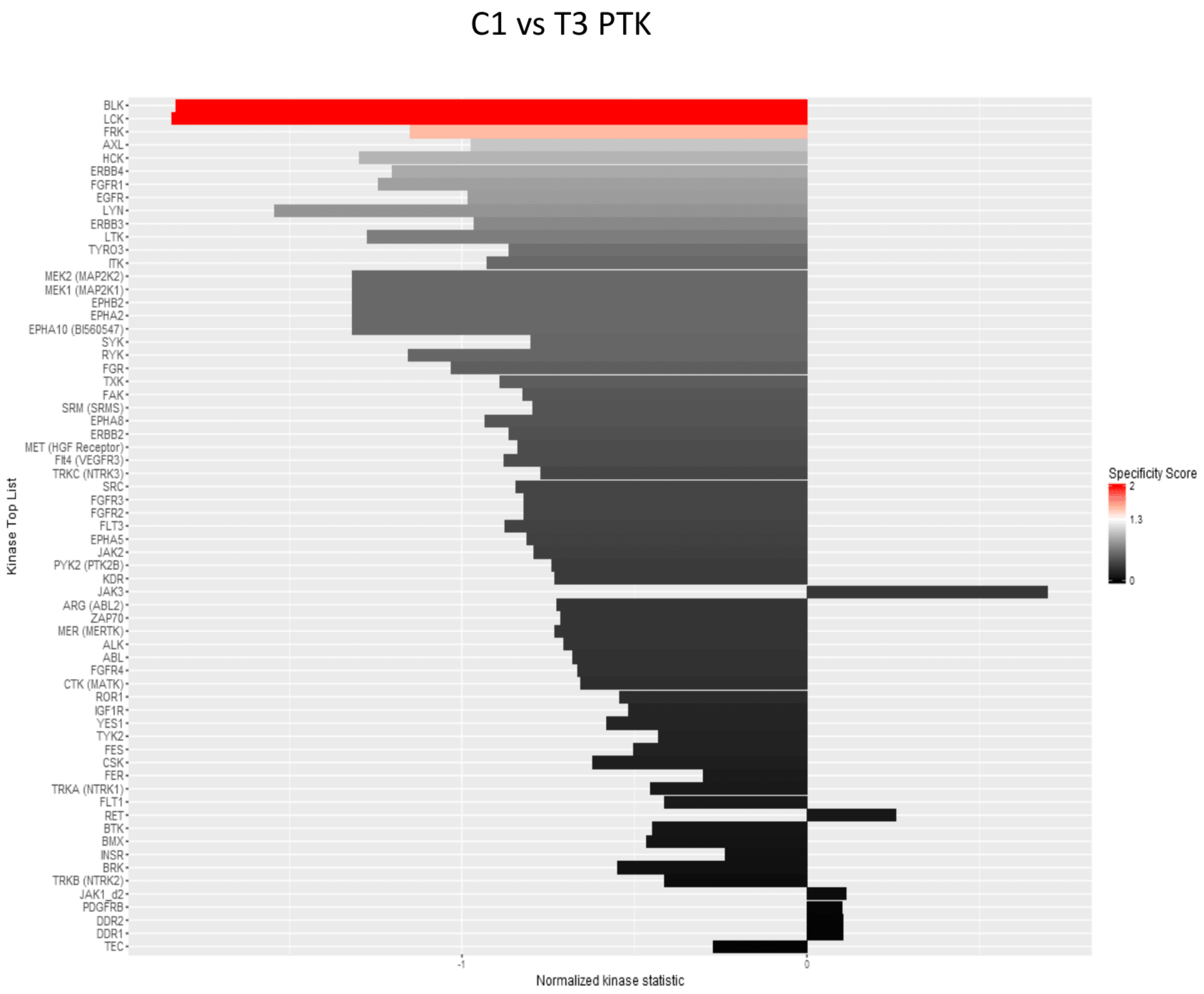

Figure S7. Complete list of up- or downregulated tyrosine kinases of the third tumor from the treatment group (T3) in comparison with the first HCC-1 xenograft tumor of the control group (C1) after profiling using PamGene assays as described in Figure 3I

Figure 2A

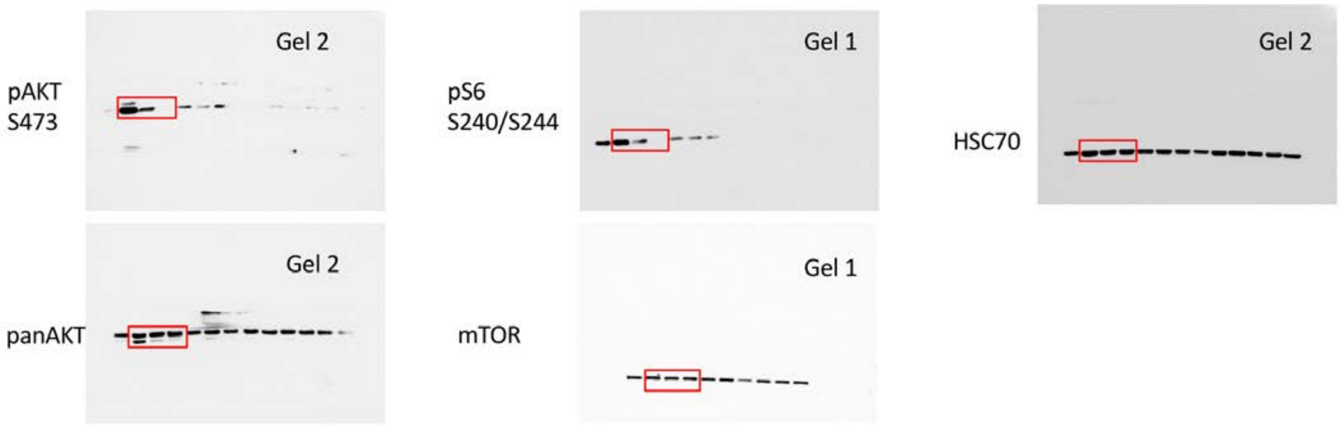

Gel 1

1	2	3	4	5	6	7	8	9	10
Huh7-luc	Hep3B	HCC1 DMSO in vitro	HCC1 MKRAD in vitro	HCC1 placebo in vivo	HCC1 placebo in vivo	HCC1 placebo in vivo	HCC1 treatment in vivo	HCC1 treatment in vivo	HCC1 treatment in vivo

Gel 2

1	2	3	4	5	6	7	8	9	10	11	12	13
Huh7-luc	Hep3B	HCC1 DMSO	HCC1 MKRAD	HCC1 placebo	HCC1 placebo	HCC1 placebo	HCC1 treatment	HCC1 treatment	HCC1 treatment	HeLa	MDA-MB-231	HEP3B

Figure 2C

Gel 1

1	2	3	4	5	6	7	8	9	10	11	12	13	14
Hep3B	Huh7-luc	SK-HEP1 DMSO	SK-HEP1 DMSO	SK-HEP1 MK2206	SK-HEP1 MK2206	SK-HEP1 MK2206	SK-HEP1 RAD001	SK-HEP1 RAD001	SK-HEP1 RAD001	SK-HEP1 MK/RAD	SK-HEP1 MK/RAD	SK-HEP1 MK/RAD	SK-HEP1 DMSO

Gel 2

1	2		3	4	5	6	7	8	9	10	11	12	13	14
Hep3B	Huh7-luc		SK-HEP1 DMSO	SK-HEP1 DMSO	SK-HEP1 DMSO	SK-HEP1 MK2206	SK-HEP1 MK2206	SK-HEP1 MK2206	SK-HEP1 RAD001	SK-HEP1 RAD001	SK-HEP1 RAD001	SK-HEP1 MK/RAD	SK-HEP1 MK/RAD	SK-HEP1 MK/RAD

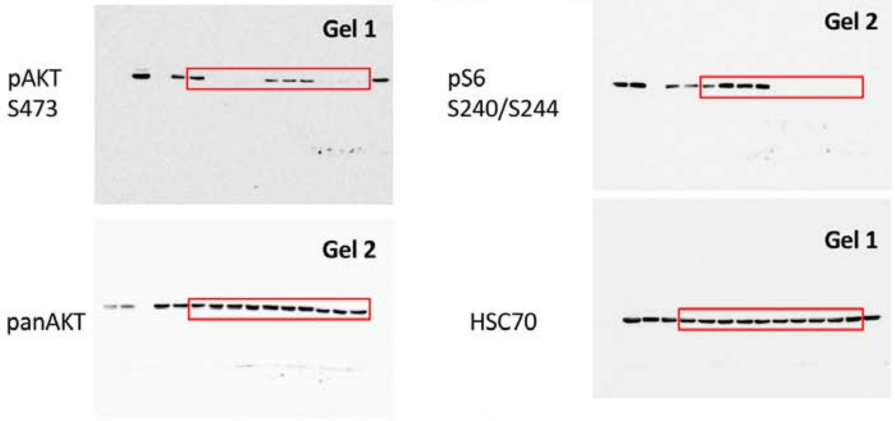

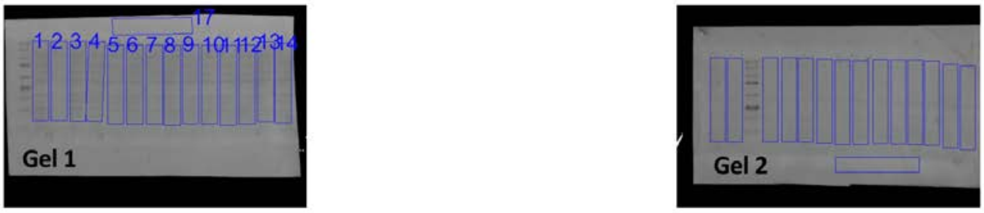

Figure 3B

1	2	3	4	5	6	7	8	9	10	11	12	13	14
HCC1 Staurospori n	HCC1	HCC1 DMSO	HCC1 RAD	HCC1 MK2206	HCC1 MK/RAD	HCC1 DMSO	HCC1 RAD	HCC1 MK2206	HCC1 MK/RAD	HCC1 DMSO	HCC1 RAD	HCC1 MK2206	HCC1 MK/RAD

pAKT S473

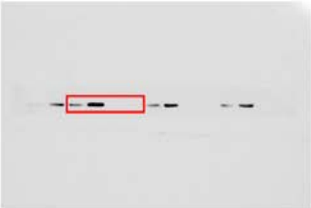

pS6 S240/S244

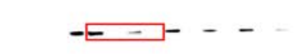

S6

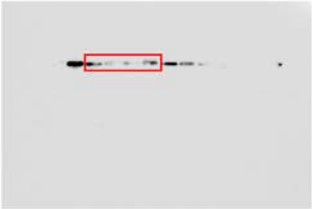

panAKT

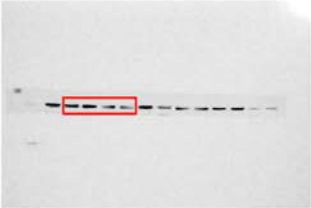

HSC70

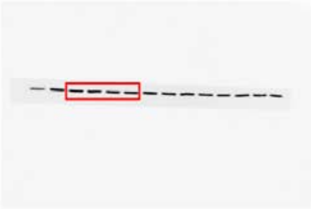

pGSK3a/ß S9

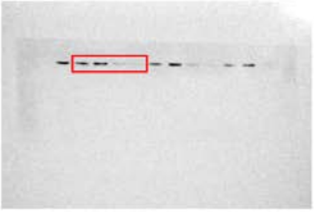

mTOR

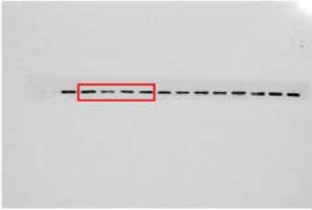

Figure 3E

pAKT S473

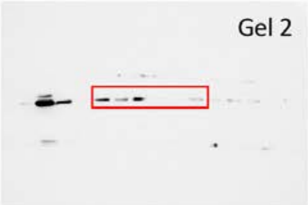

pS6 S240/S244

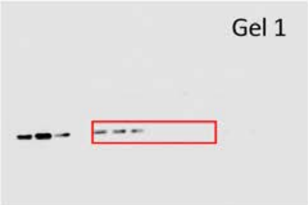

HSC70

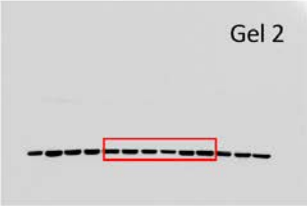

panAKT

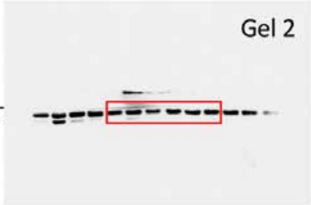

mTOR

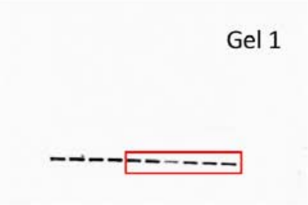

Gel 1

1	2	3	4	5	6	7	8	9	10
Huh7-luc	Hep3B	HCC1 DMSO in vitro	HCC1 MKRAD in vitro	HCC1 placebo in vivo	HCC1 placebo in vivo	HCC1 placebo in vivo	HCC1 treatment in vivo	HCC1 treatment in vivo	HCC1 treatment in vivo

Gel 2

1	2	3	4	5	6	7	8	9	10	11	12	13
Huh7-luc	Hep3B	HCC1 DMSO	HCC1 MKRAD	HCC1 placebo	HCC1 placebo	HCC1 placebo	HCC1 treatment	HCC1 treatment	HCC1 treatment	HeLa	MDA-MB-231	HEP3B

Figure 4E

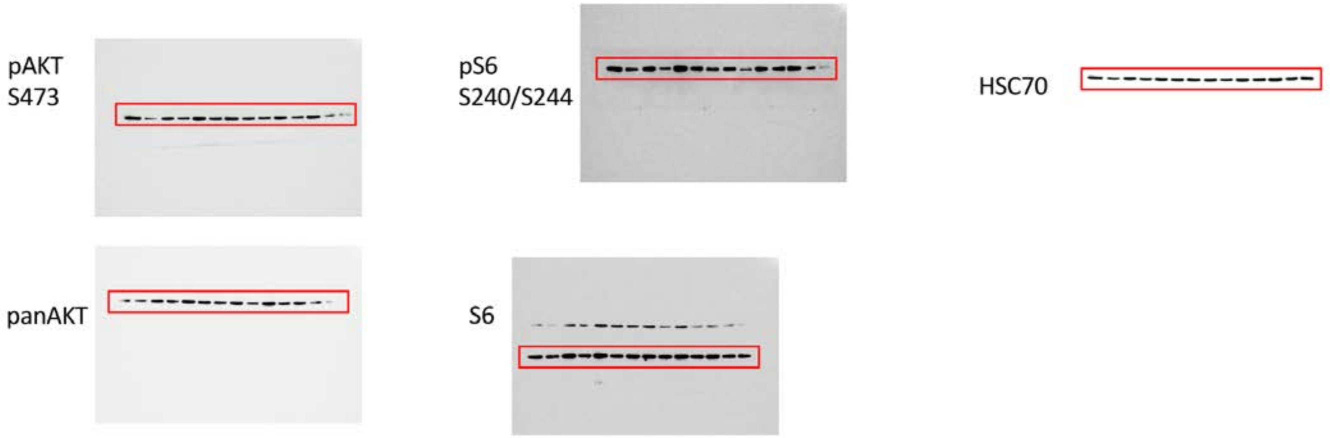

1	2	3	4	5	6	7	8	9	10	11	12	13	14
SK-HEP1 in vivo placebo #1	SK-HEP1 in vivo placebo #2	SK-HEP1 in vivo placebo #3	SK-HEP1 in vivo placebo #4	SK-HEP1 in vivo placebo #5	SK-HEP1 in vivo placebo #6	SK-HEP1 in vivo placebo #7	SK-HEP1 in vivo treatment #1	SK-HEP1 in vivo treatment #2	SK-HEP1 in vivo treatment #3	SK-HEP1 in vivo treatment #4	SK-HEP1 in vivo treatment #5	SK-HEP1 in vivo treatment #6	SK-HEP1 in vivo treatment #7

1	2	3	4	5	6	7	8	9	10	11	12	13	14
Huh7 Staurosporin	Huh7	Huh7 DMSO	Huh7 RAD	Huh7 MK2206	Huh7 MK/RAD	Huh7 DMSO	Huh7 RAD	Huh7 MK2206	Huh7 MK/RAD	Huh7 DMSO	Huh7 RAD	Huh7 MK2206	Huh7 MK/RAD

Figure 5A

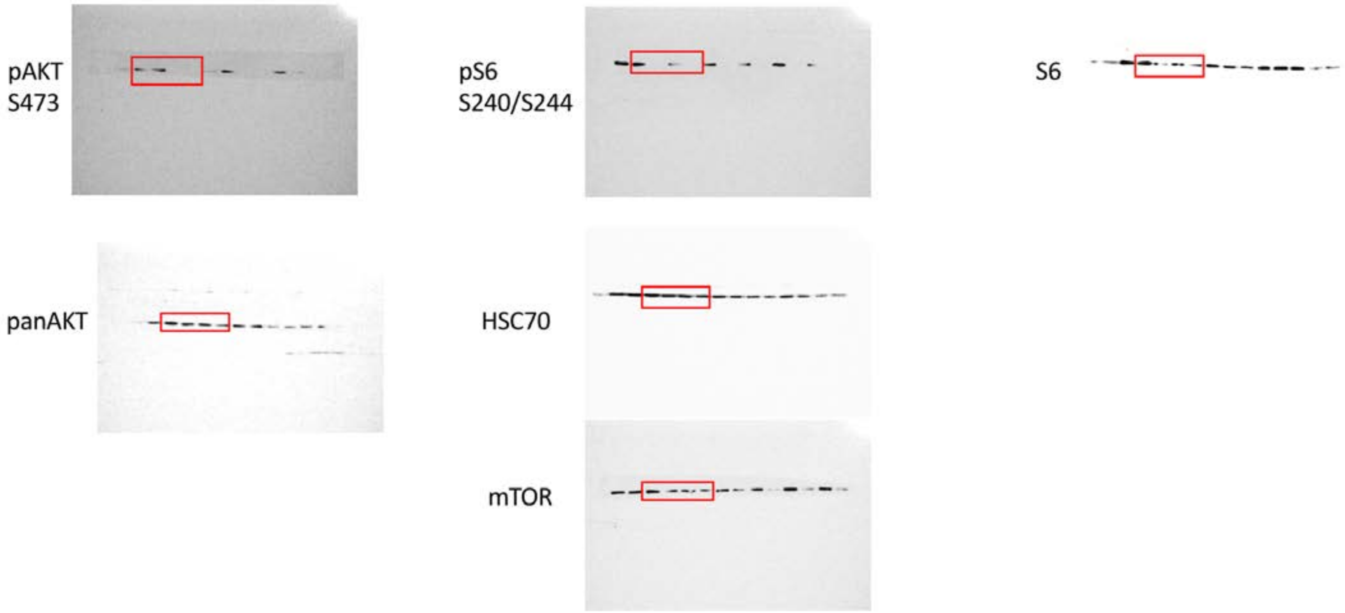

Figure 5D

1	2	3	4	5	6	7	8	9	10	11	12	13	14
Placebo Huh7 in vivo #32	Placebo Huh7 in vivo #34	Placebo Huh7 in vivo #35	Placebo Huh7 in vivo #36	Placebo Huh7 in vivo #53	Placebo Huh7 in vivo #55	Treatment Huh7 in vivo #37	Treatment Huh7 in vivo #39	Treatment Huh7 in vivo #40	Treatment Huh7 in vivo #41	Treatment Huh7 in vivo #47	Treatment Huh7 in vivo #52	DMSO	MDA-MB-231

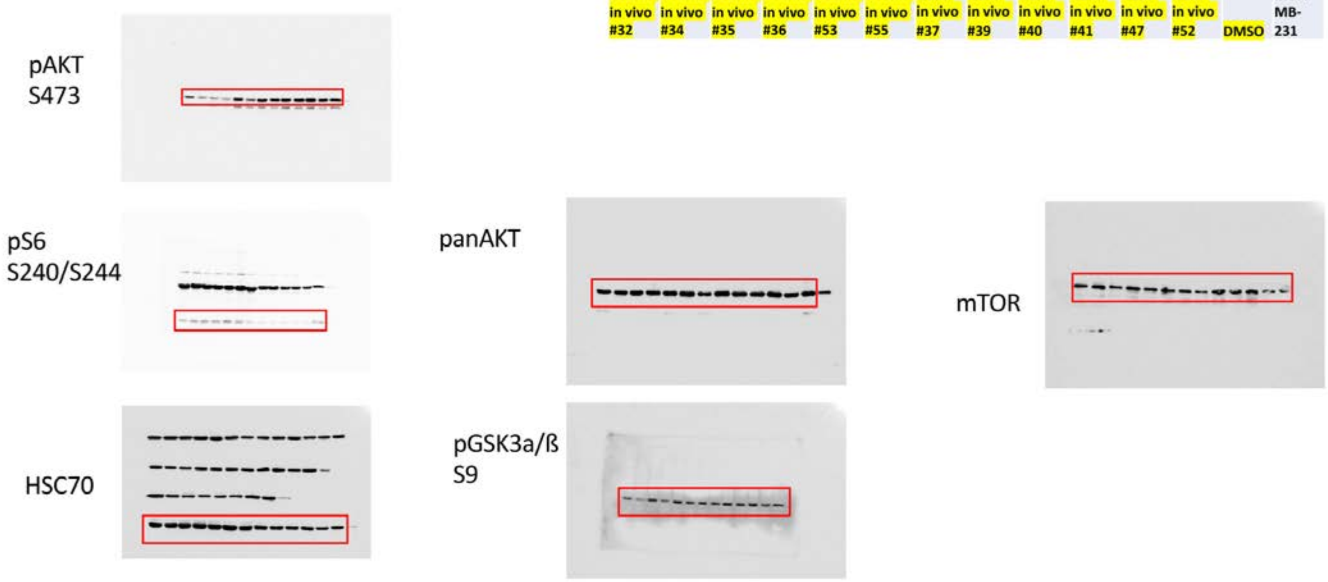

Figure 5E

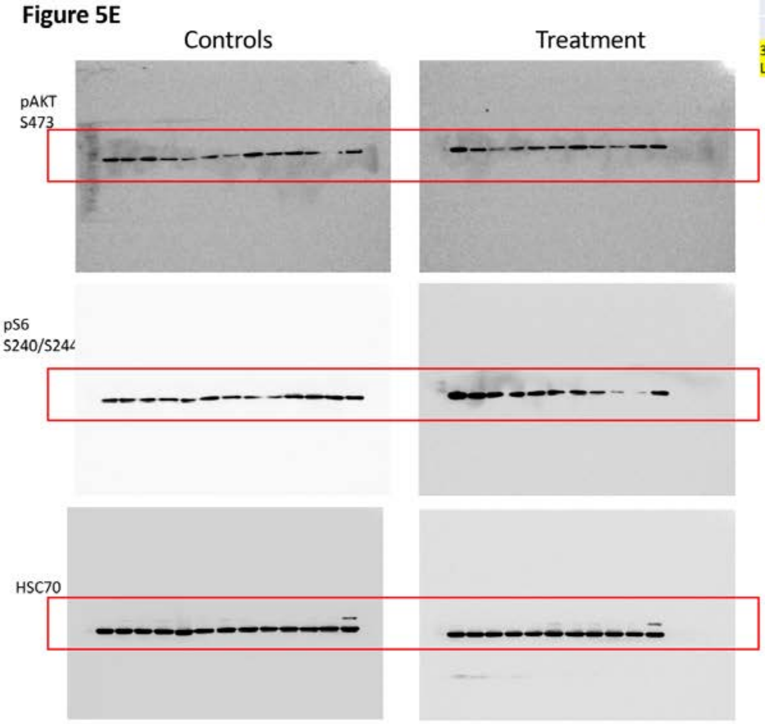

Controls:

1	2	3	4	5	6	7	8	9	10	11	12	13	14
31 LLL	32 LML	31 RML	32 RML	33 RML	34 RML	35 RML	43 RML	44 RML	53 RML	54 RML	55 RML	DMSO	MDA-MB-231 *

* only on panAKT blots)

Treatment:

1	2	3	4	5	6	7	8	9	10	11	12
37 RML	38 RML	39 RML	40 RML	41 RML	42 RML	46 RML	47 RML	48 RML	52 RML	DMSO	MDA-MB-231 *

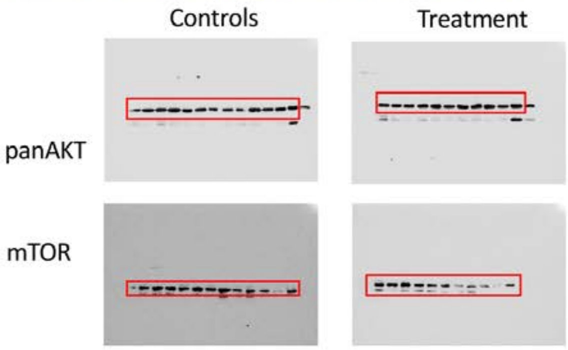

Figure 5F

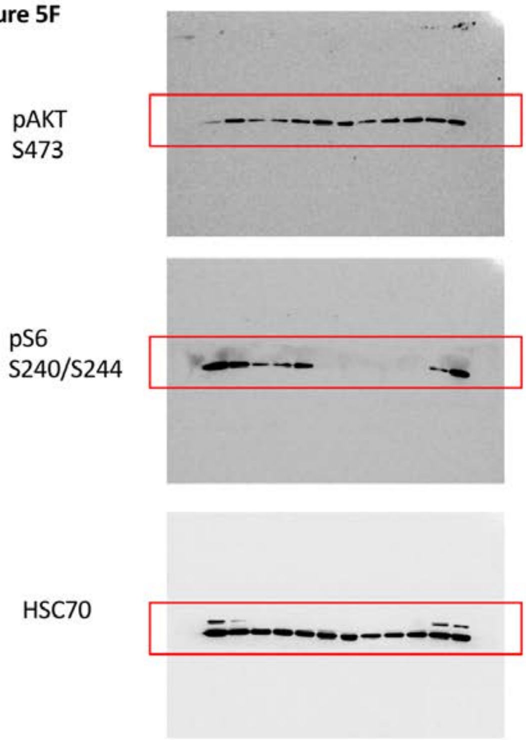

1	2	3	4	5	6	7	8	9	10	11	12
35 lymph node	31 gut	32 gut	33 spleen	43 spleen	37 sternal	38 sternal	40 sternal	41 sternal	42 sternal	40 lymph node	DMSO

Figure S8. Original uncropped Western blots.
